# Linking Bacterial Endophytic Communities to Essential Oils: Clues from *Lavandula angustifolia* Mill

**DOI:** 10.1155/2014/650905

**Published:** 2014-05-26

**Authors:** Giovanni Emiliani, Alessio Mengoni, Isabel Maida, Elena Perrin, Carolina Chiellini, Marco Fondi, Eugenia Gallo, Luigi Gori, Valentina Maggini, Alfredo Vannacci, Sauro Biffi, Fabio Firenzuoli, Renato Fani

**Affiliations:** ^1^Trees and Timber Institute, National Research Council, Via Madonna del Piano, No. 10, Sesto Fiorentino, 50019 Florence, Italy; ^2^Laboratory of Microbial and Molecular Evolution, Department of Biology, University of Florence, Via Madonna del Piano 6, Sesto Fiorentino, 50019 Florence, Italy; ^3^Department of Neuroscience, Psychology, Drug Research and Child Health, University of Florence, Viale Pieraccini 6, 50139 Florence, Italy; ^4^Center for Integrative Medicine, Careggi University Hospital, University of Florence, Viale Pieraccini 6, 50139 Florence, Italy; ^5^Il giardino delle Erbe, via Del Corso 6, Casola Valsenio, 48010 Ravenna, Italy

## Abstract

Endophytic bacteria play a crucial role in plant life and are also drawing much attention for their capacity to produce bioactive compounds of relevant biotechnological interest. Here we present the characterisation of the cultivable endophytic bacteria of *Lavandula angustifolia* Mill.—a species used since antiquity for its therapeutic properties—since the production of bioactive metabolites from medical plants may reside also in the activity of bacterial endophytes through their direct production, PGPR activity on host, and/or elicitation of plant metabolism. Lavender tissues are inhabited by a tissue specific endophytic community dominated by Proteobacteria, highlighting also their difference from the rhizosphere environment where Actinobacteria and Firmicutes are also found. Leaves' endophytic community resulted as the most diverse from the other ecological niches. Overall, the findings reported here suggest: (i) the existence of different entry points for the endophytic community, (ii) its differentiation on the basis of the ecological niche variability, and (iii) a two-step colonization process for roots endophytes. Lastly, many isolates showed a strong inhibition potential against human pathogens and the molecular characterization demonstrated also the presence of not previously described isolates that may constitute a reservoir of bioactive compounds relevant in the field of pathogen control, phytoremediation, and human health.

## 1. Introduction

A diverse range of bacteria, including pathogens, mutualists, and commensals is supported by plants. These bacteria grow in and around roots, in the vasculature, and on aerial tissues [1, 2]. In particular, endophytic bacteria can be defined as those bacteria that colonize the internal tissue of the plants with no external sign of infection or negative effect on their host [2, 3]. It is increasingly evident that bacterial endophytes influence plant physiology, facilitating the uptake of nutrients such as nitrogen, phosphorus, sulphur, magnesium, and calcium [4] and showing plant growth-promoting activity (PGPR) related to the production of phytohormones involved in regulatory metabolism, such as ethylene [5], indole-3-acetic acid (IAA), and acetoin, 2,3-butanediol, [6–8]. Moreover, endophytic bacteria can also improve plant growth via nitrogen fixation [9]. Other relevant functions performed by endophytic bacteria are represented by the decrease or prevention of the pathogenic effects of certain parasitic microorganisms with the production of antimicrobial compounds or by increasing plant tolerance to pollution or stresses [3, 10]. Bacterial endophytes are drawing increasing interest as together with fungal endophytes they are reported to produce a number of bioactive metabolites relevant to human health, such as antibiotics [11], antitumor compounds [12, 13], and anti-inflammatory agents [14].

Endophytic bacteria can be further classified as “obligate” or “facultative” in accordance with their life strategies. Obligate endophytes are strictly dependent on the host plant for their growth and survival, besides, transmission to other plants could occur only by seeds or via vectors, while facultative endophytes could grow outside host plants [15]. Several studies have shown that facultative endophytes constitute the largest fraction of the endophytic bacterial communities [5]; in fact large plant-by-plant (both at interspecific and intraspecific level) differences in the bacterial communities composition have been found [16–20], supporting the idea that the ability to enter plant tissues is a widespread phenotype of soil and rhizosphere bacteria, and that plants exert only a limited selectivity on the colonizing bacterial communities [21], even if clues for a control over the bacterial colonizers operated by yet not completely clarified plant mechanism(s) are reported [5, 22]. Actually, a recent bioinformatic study has shown that inside the class of Alphaproteobacteria (which includes well known bacterial endophytes, such as rhizobia and methylobacteria) few genomic signatures only could distinguish endophytes from nonendophytes [23]. Another common feature of endophytic bacterial community is their strong temporal and spatial variability [16, 19, 24]; the community composition not only varies in the soil compartment between the rhizosphere and the root internal tissues in response to complex and not yet fully clarified biotic (plant species and genotype dependent) and abiotic (soil characteristics) driven processes [16, 24], but also among different aerial tissues (stems, leaves, flowers) of the same plant [25, 26]. Total endophytes are influenced not only by the location within the plant but also by the presence of the main components of the essential oil in the leaves of aromatic plants [27].

However, all the studies performed so far have analysed mainly the bacterial communities in terms of species composition (or higher taxonomic ranks), especially using cultivation-independent techniques, as 16S rRNA gene libraries or metagenetic sequencing [20, 28, 29]. While cultivation-independent techniques allow a deep coverage of taxonomic diversity of bacterial communities, they provide only partial information on community structure and clearly hamper the possibility to test the actual presence of PGPR activities and bioactive molecule production in the bacterial community.

Among crops, medicinal plants are stirring much attention due to the increasing demand for green chemistry approaches, sustainable practices, and especially in the quest for novel antibiotics able to tackle the increasing multidrug resistance of pathogenic bacteria. In spite of the high relevance of medicinal plants, to the best of our knowledge, very little, if nothing at all, is known about the endophytic bacterial communities isolated from medicinal plants. For this reason, in this work we isolated and preliminary characterized from a taxonomical viewpoint the aerobic heterotrophic cultivable endophytic bacterial community of lavender (*Lavandula angustifolia* Mill.syn.* Lavandula officinalis* Chaix,* Lavandula vera* DC,* Lavandula spica L. var angustifolia* Auct.). Lavender has a long history of medicinal use and is purported to possess relaxant, neurological, and antibacterial effects [30].

The essential oil of Lavender has been used since antiquity for a variety of medical application [31]; in particular anxiolytic activity of Lavender oil was confirmed [32] and its antimicrobial activity was demonstrated against different pathogens [33]. Moon et al. [34] reported that low (≤1%) concentrations of* L. angustifolia* and* L. × intermedia* oil can completely eliminate* Giardia duodenalis*,* Trichomonas vaginalis,* and* Hexamita inflata in vitro*. Preliminary results also highlighted the possibility for lavender essential oil to be used as antibiotic resistance modifying agent in microorganisms [35].

It has also been demonstrated that the production of bioactive compounds is significantly impacted by the genetic milieu of the plant and by environmental factors [31]; in this framework, it is increasingly evident that the collection of microorganism living in association with complex organisms, generally defined as microbiota, plays a fundamental role in shaping their phenotypic features. Since the qualiquantitative production of bioactive metabolites in medical plants may reside also in the activity of bacterial endophytes through the direct production, PGPR activity, and/or elicitation of plant metabolism [36], the purpose of this research was therefore to perform a cultivation-dependent study of the mesophilic aerobic heterotrophic bacterial endophytic community of the relevant medical and balsamic species* L. angustifolia* in order to (i) identify its composition, (ii) its diversity between different plant compartments (roots, stems, and leaves), and (iii) its diversity in relation to the rhizosphere bacterial community, and (iv) build a collection of isolates to be screened for the production of bioactive compounds, and (v) test a panel of randomly selected endophytic bacteria* versus* opportunistic human pathogens belonging to the* Burkholderia cepacia* complex (Bcc). We have chosen these bacteria since many strains of the complex are opportunistic human pathogens and represent a serious concern for cystic fibrosis (CF) patients and immunocompromised individuals [37], responsible for the “cepacia syndrome,” characterized by high fever, severe progressive respiratory failure, leukocytosis, and elevated erythrocyte sedimentation rate. Moreover, Bcc strains are (naturally) resistant to many antibiotics such as cephalosporin, *β*-lactams, polymyxins, and aminoglycosides; therefore, Bcc infections are very problematic to eradicate [37, 38]. In spite of this high degree of resistance to many antibiotics, it has been recently demonstrated that essential oils extracted from six medicinal plants are able to completely inhibit the growth of Bcc members, including those with a clinical origin and exhibiting resistance to many antibiotics [39].

## 2. Materials and Methods

### 2.1. Plant Sampling and Isolation of Mesophilic Cultivable Endophytic and Rhizosphere Bacteria

Five agamically propagated potted* L. angustifolia* plants were collected from the “Giardino delle Erbe,” located in Casola Valsenio, Italy, in June 2012. On the same day, plants were transported to the laboratory for processing. Pieces of 200 mg of fresh leaves, stems, and roots tissues were collected from each of the 5 plants and bulked (for a final sample of 1 g) to account for plants to plants variability. Roots and stems samples were divided into 1 cm long pieces and surface-sterilized for 40 s with 70% ethanol followed by 10 min with 2.5% sodium hypochlorite. Plant leaves were surface-sterilized for 20 s with 70% ethanol followed by 5 min with 2.5% sodium hypochlorite. To remove the disinfectant, sections were rinsed three times for 5 min in sterile distilled water. Samples were then dried with sterile filter paper and subsequently ground with 2 mL 0.9% sodium chloride with a sterile mortar. Aliquots (100 *μ*L) of the last washing water were plated in triplicate as sterility controls. Samples (100 *μ*L) of tissue extracts and their different dilutions were plated in triplicate. Aliquots of 200 mg of soil from each pot were collected and bulked for 1 g total. The soil samples were then resuspended in 5 mL of 10 mM Mg_2_SO_4_ and placed under stirring for 1 h at room temperature.

Endophytic and rhizospheric bacteria were grown in triplicate on solid 5% tryptone soya broth (TSB) medium (Oxoid Ltd., Basingstoke, Hampshire, UK) at 30°C for 72 h. The number of aerobic heterotrophic bacteria was determined as colony-forming units (CFUs). Each CFU determination was performed in triplicate and an average value of bacterial titre was determined. From each sample, colonies were randomly selected and singularly plated on 5% solid TSB Petri dishes. From each isolate, glycerol stock (25% final concentration) was prepared and stored at −80°C.

### 2.2. PCR Amplification and Sequencing of 16S rRNA Coding Genes from Bacterial Endophytes

Cell lysates were prepared by dissolving a bacterial colony in 100 *μ*L sterile distilled water and incubation at 99°C for 10 min, followed by 5 min at 4°C. An aliquot of 2 *μ*L lysate was used for the amplification reaction by polymerase chain reaction (PCR). Amplification of 16S rRNA genes was performed in a total volume of 20 *μ*L containing 2 *μ*L of 10X reaction buffer (Polymed, Firenze, Italy), 1.5 mM MgCl_2_, 10 pmol of each primer [27f, 5′-GAGAGTTTGATCCTGGCTCAG, and 1495r, 5′-CTACGGCTACCTTGTTACGA], 0.25 mM of dNTP mix, 2 U of* Taq* DNA polymerase (Polymed). PCR reaction conditions were as described by Mengoni et al. [40].

For sequencing reaction, amplified 16S rDNA fragments were excised from 1% agarose gels and purified using the QIAquick Gel Extraction (Qiagen) according to the manufacturer's instructions. Direct sequencing of amplicons was performed at the Genechron laboratory (Ylichron Srl, Italy) with primer 27f on an ABI3730 DNA analyser (Applied Biosystems, Foster City, CA, USA) using the Big Dye Terminator Kit.

### 2.3. Sequence Analysis and Phylogenetic Tree Construction

The sequences presented in this work have been deposited in GeneBank database under the accession numbers KF202531-KF202915. Partial 16S rDNA sequences were matched against nucleotide sequences available in GenBank database using the BLASTn program [41].

MUSCLE [42] (http://www.drive5.com/muscle) was used to align the 16S rDNA sequences obtained with the most similar orthologous sequences retrieved from the Ribosomal Database Project (RDP) database (http://rdp.cme.msu.edu/). Alignments were trimmed to eliminate poorly aligned region and used to build Bayesian, Maximum Parsimony (MP), and the Neighbor joining (NJ) dendrograms.

Bayesian dendrograms were obtained with MrBayes 3.2 [43] with GTR substitution model with gamma-distributed rate variation across sites with 1000000 generations. MP dendrograms were obtained with MEGA 5 [44] (http://www.megasoftware.net/) using the Tree-Bisection-Regrafting (TBR) algorithm with search level 3 in which the initial trees were obtained by the random addition of sequences (500 replicates); the robustness of the inferred trees was evaluated by 1000 bootstrap resamplings; consistency and retention indexes (CI and RI) were calculated with Mesquite software 2.75 (http://mesquiteproject.org). NJ dendrograms were obtained after calculation of a Kimura two-parameter distance matrix with the software Mega 5. The robustness of the inferred trees was evaluated by 1000 bootstrap resamplings.

### 2.4. Statistical Analysis

Pairwise sequence identity values (not taking deletion into account) were calculated using the stand-alone Clustal Omega [45]. Genetic distances among OTU were calculated using the Kimura 2 parameter model and 1000 bootstraps replications in the MEGA 5.2.1 software [44]. Diversity indexes were calculated with the PAST3 software [46]. Pairwise differentiation (Fst) values were calculated inside the GenoDive v 2.0b25 software (http://www.patrickmeirmans.com/software/GenoDive.html), using vector of presence/absence of the bacterial genera in the different samples with 999 permutations.

### 2.5. Cross-Streaking Experiments

Antibacterial activity was determined by using the cross-streak method [47, 48]. Hereinafter, endophytic bacterial isolates to be tested for inhibitory activity will be termed “tester” strains, whereas Bcc strains used as a target will be called “target” strains. Cross-streaking experiments were carried out as previously described [47] by using Petri dishes. Tester strains were streaked across one-half of an agar plate with PCA medium and incubated at 30°C. After 2 days of incubation, target strains were streaked on PCA medium perpendicular to the initial streak and plates were further incubated at 30°C for 2 days. The antagonistic effect was indicated by the failure of the target strains to grow. The list of target Bcc strains used in this work is reported in Table 3.

## 3. Results

### 3.1. Composition of Endophytic Bacterial Communities Isolated from* L. Angustifolia*

Aerobic heterotrophic culturable bacteria were isolated from leaves, stems, and roots of 5 plants of* Lavandula angustifolia*. Leaves samples had the highest number of CFU/g fresh weight (6.8 × 10^5^), whereas roots had the lowest values (1.6 × 10^4^), the difference being anyway of one order of magnitude only (stem and rhizospheric soil showed titers of 6.5 × 10^4^ and 3.4 × 10^5^, resp.). Plates were visually inspected and no increase in colony number was observed with an extended incubation time of up to 7 days.

On the triplicate plates of the same plant portion, 100 colonies were randomly selected and isolated on TSA agar plates. A collection of 400 colonies was then prepared with isolates from the four samples types, namely, rhizospheric soil, roots, stems, and leaves.

In order to determine the taxonomic composition of the bacterial communities isolated from the different compartments of* L. angustifolia* plants and from rhizospheric soil, 16S rRNA genes were PCR-amplified from the isolates of the collection as described in Materials and Methods. An amplicon of the expected size was obtained from each bacterial isolate (data not shown) and the nucleotide sequence of the amplicons was then determined. In this way we obtained 395 sequences, each of which was used as seed to probe databases.

**Figure 1:**
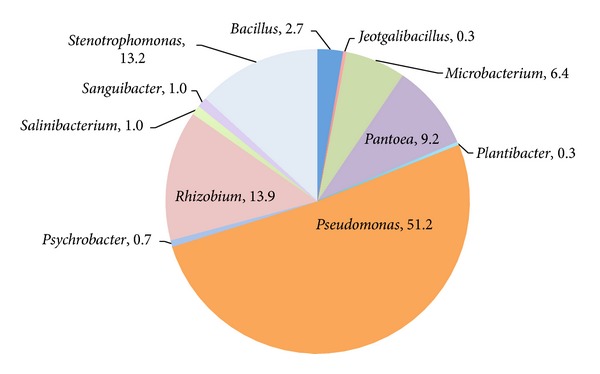
Composition of the aerobic heterotrophic endophytic (*sensu stricto*) bacterial community in* L. angustifolia* tissues. The composition is reported as percentages of the total number of characterized isolates (*n* = 395).

Table 1 reports the percentage distribution of the identified bacterial phyla in the different plant compartments and in the rhizospheric soil, along with standard diversity indexes calculated on genera distribution; the results show a relative peak of diversity in the leaf and a minimum in the stem (Shannon index 2.29 and 1.06, resp.).  Figure 1 depicts the taxonomic composition of the total lavender aerobic heterotrophic cultivable endophytic community made up by a total of 11 genera; the majority of the isolated strains (88%) belonged to proteobacteria, with a dominance of gammaproteobacteria (74%),* Pseudomonas* being the most abundant genus accounting for the 51% of total community, followed by* Stenotrophomonas* (13%) and* Pantoea* (9%).* Rhizobium* is also frequently found in the internal tissue of lavender, representing 14% of the total collection. Actinomycetales are also represented (8%) with the genus* Microbacterium* being the most abundant (6%). Other genera were found, namely,* Bacillus*,* Plantibacter*,* Psychrobacter*,* Sanguibacter*,* Salinibacterium*, and* Jeotgalibacillus *representing collectively 6% of the endophytic collection.

**Figure 2:**
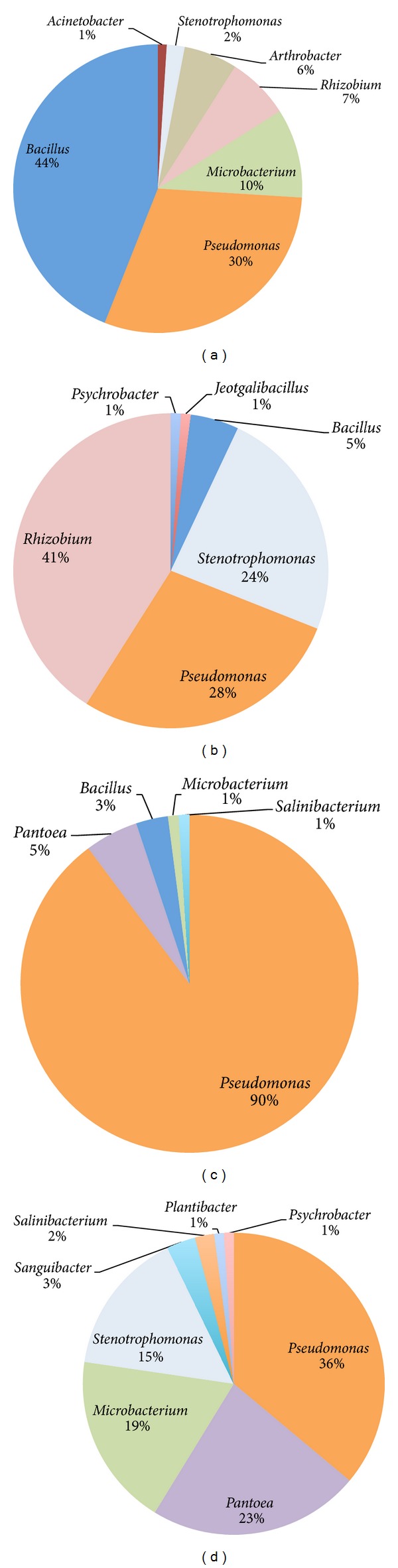
Composition of the aerobic heterotrophic bacterial community of* L. angustifolia* rhizospheric soil (a), roots (b), stem (c), and leaves (d). The composition is reported as percentages of the total number of characterized isolates for each sample.

Bacteria isolated from the rhizospheric soil showed a quite different taxonomic composition (Figure 2(a)) when compared to the overall endophytic community; in the rhizosphere,* Bacillus* is the most abundant genus (44%), followed by* Pseudomonas* (30%),* Microbacterium* (10%),* Rhizobium* (7%), and* Arthrobacter* (6%), a genus that is not detected in the endophytic community.  Figure 2 shows also the composition of the endophytic communities isolated from the different tissues (panels (b), (c), and (d), for roots, stem, and leaves, resp.); the three compartments are characterized by strikingly different communities, for the differential presence of genera (e.g.,* Bacillus *is found in roots and stem, but absent in the leaves,* Sanguibacter* and* Plantibacter* are detected in the leaves only,* Jeotgalibacillus *is found only in the roots) and the overall diversity (8 genera are found in the leaves and only 5 genera are in the stem) and the different relative presence of the other most abundant genera.

**Figure 3:**
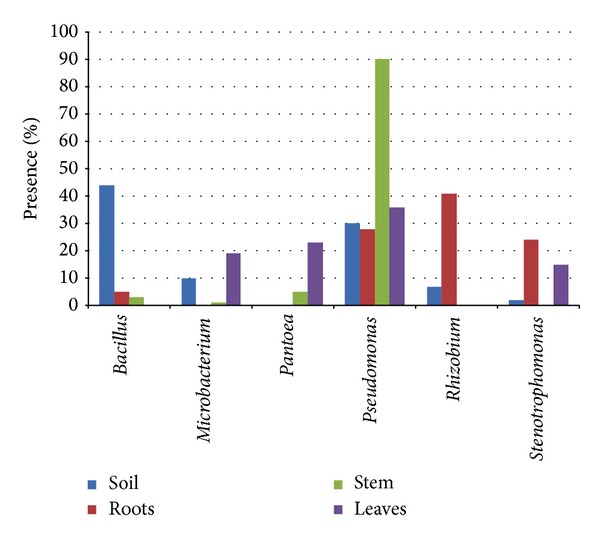
Comparison of the relative abundance of bacterial genera in the different samples; only genera with percentages >5% in at least one of the samples are reported.

Figure 3 shows the distribution of main genera (occurring in >5% of total isolates) in the 3 lavender tissues and in its rhizosphere. Isolates belonging to the genus* Bacillus* decrease their abundance from the rhizosphere to the stem and disappear in the leaves, while* Pantoea* and* Microbacterium *show a similar distribution in stems and leaves, but are both absent in the roots. Isolates from the* Pseudomonas* genus dominate in the stem community and are similarly occurring (ca. 30% of total) in the other 3 samples.* Rhizobium* spp. was found in soil and represented 40% of roots isolates. Lastly,* Stenotrophomonas* spp. is abundant in the roots and leaves, but absent in the stem. To further analyse the diversity of the bacterial communities isolated, pairwise differentiation (Fst) values were calculated on vector of presence/absence of bacterial genera; the lowest differentiation was registered among roots and rhizospheric soil (Fst = 0.198) communities, while the highest among stems and leaves (Fst = 0.512). Overall, the stem endophytic community resulted as the most differentiated from the others (Fst = 0.428 and 0.394 versus roots and rhizospheric soil, resp.) while the leaves community showed similar differentiation values when compared to rhizospheric soil (Fst = 0.245) and roots (Fst = 0.239).

**Figure 4:**
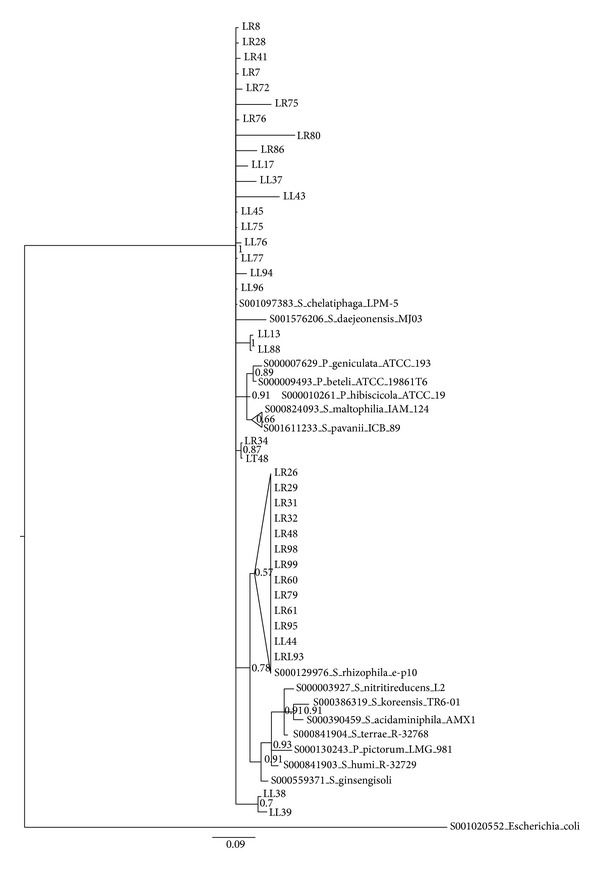
Bayesian dendrogram showing the relationships among the 16S rDNA sequences of 37 isolates belonging to the genus* Stenotrophomonas* and those of reference type strains. Posterior probability values are indicated at the node. Nodes are collapsed at 70% probability. LL = bacteria isolated from the leaves, LR = bacteria isolated from the roots (see Section 2 for details).

**Figure 5:**
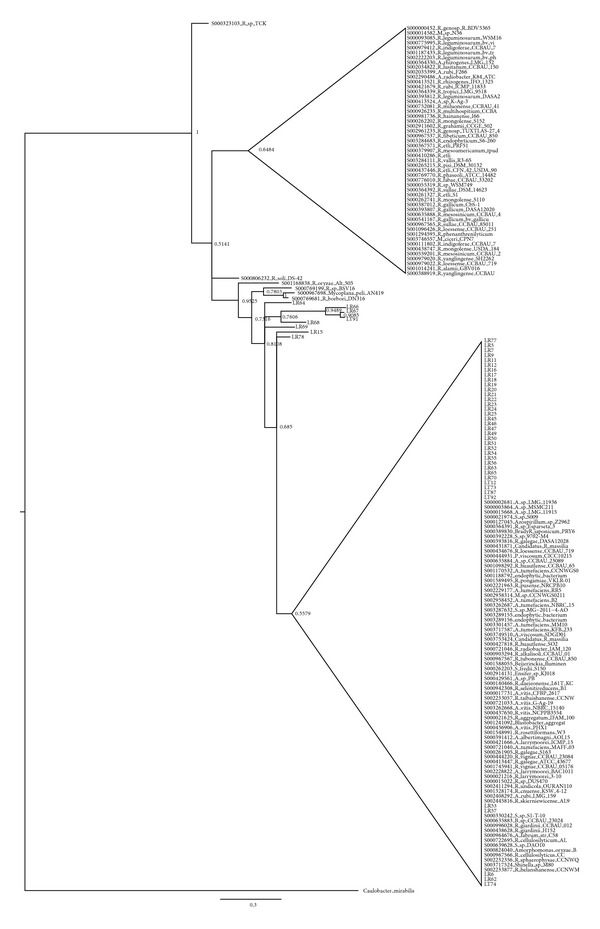
Bayesian dendrogram showing the relationships among the 16S rRNA gene sequences of 46 isolates belonging to the genus* Rhizobium* and those of reference type strains. Posterior probability values are indicated at each node. Nodes are collapsed at 70% probability. LT = bacteria isolated from the rhizosphere, LR = bacteria isolated from the roots (see Section 2 for details).

**Figure 6:**
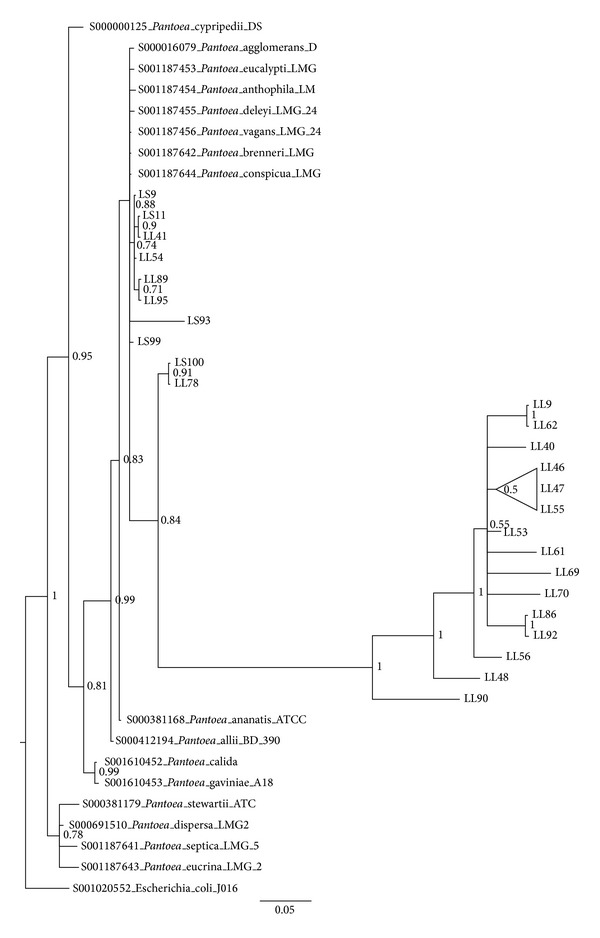
Bayesian dendrogram showing the relationships among the 16S rRNA gene sequences of 25 isolates belonging to the genus* Pantoea *and those of reference type strains. Posterior probability values are indicated at the node. Nodes are collapsed at 70% probability. LS = bacteria isolated from the stem, LL = bacteria isolated from the leaves (see Section 2 for details).

**Figure 7:**
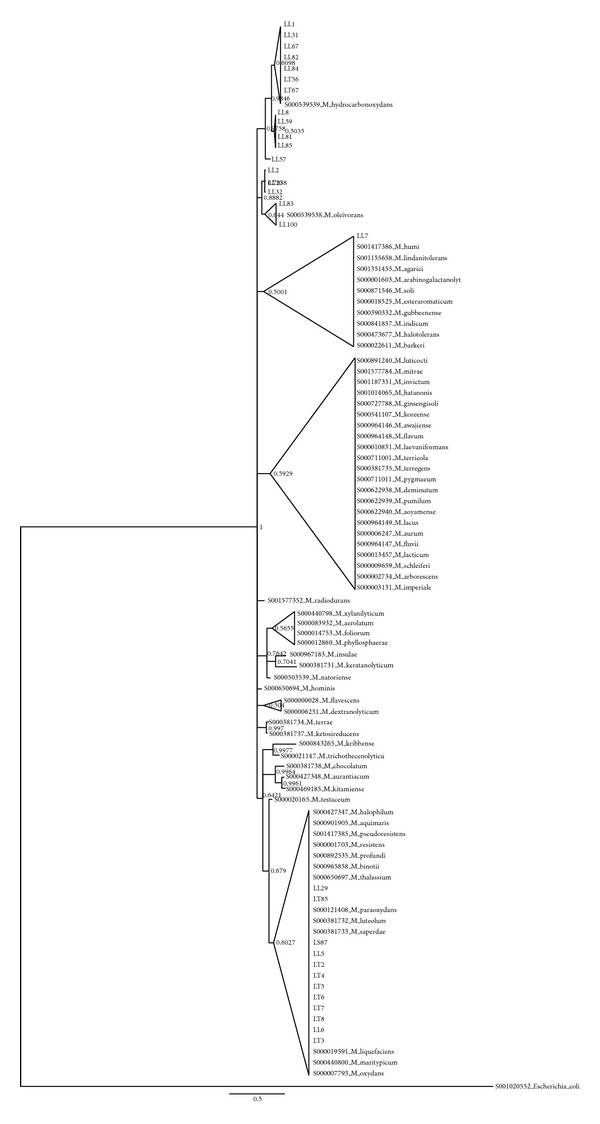
Bayesian dendrogram showing the relationships among the 16S rRNA gene sequences of 30 isolates belonging to the genus* Microbacterium* and those of reference type strains. Posterior probability values are indicated at the node. Nodes are collapsed at 70% probability. LT = bacteria isolated from the rhizosphere, LS = bacteria isolated from the stem, LL = bacteria isolated from the leaves (see Section 2 for details).

Considering the low number of bacterial phyla found in the endophytic community (>95% of isolates were assigned to only 6 genera), we analysed the intrageneric level of diversity by means of 16S rRNA gene sequence identity values. Data obtained are shown in Table 2. From the 16S rRNA gene sequence diversity indices reported that the isolates assigned to the genus* Rhizobium* possessed the lowest overall diversity, suggesting the presence of a low number of species belonging to the genus* Rhizobium*. On the other extreme, the* Pantoea* isolates showed a stronger differentiation supporting the idea of the presence of different species (sequence identity < 97%). To further characterize the isolated endophytic and rhizosphere bacteria, Bayesian, Maximum Parsimony and Neighbor Joining trees based on 16S rRNA genes were constructed for the most abundant genera, namely,* Stenotrophomonas* (Figure 4 and Suppl. Figures 3 and 9  in Supplementary Material available online at http://dx.doi.org/10.1155/2014/650905),* Rhizobium* (Figure 5 and Suppl. Figures 4 and 10),* Pantoea* (Figure 6 and Suppl. Figures 5 and 11),* Microbacterium* (Figure 7 and Suppl. Figures 6 and 12),* Bacillus *(Suppl. Figures 1, 7, and 13), and* Pseudomonas* (Suppl. Figures 2, 8, and 14). Overall, Bayesian and NJ dendrograms showed a stronger support (and consistency of the results) than the MP ones (see also Suppl. Table 4). The analysis of the Bayesian phylogenetic trees revealed the following.

- Endophytic bacteria isolated from the leaves and roots of lavender and assigned to the genus* Stenotrophomonas* (Figure 4) are clearly split in two groups. One group contains isolates clustering with* Stenotrophomonas chelatiphaga*; interestingly this cluster comprises isolates from both roots and leaves (no* Stenotrophomonas* were isolated from the stem), suggesting that two compartments might share bacteria belonging to the same species, even though the polymorphism shown by the aligned partial 16S sequences might suggest a diversification at the strain level. The second cluster embeds roots isolates (with the exception of leaves isolate LL44), clustering with* Stenotrophomonas rizophila*.
- Concerning rhizobia (Figure 5), a grouping in one main cluster of low differentiate sequences is present. These groups might contain new clades of plant-associated rhizobia, which deserve more investigation in the future.
- From the phylogenetic tree of Figure 6,* Pantoea* isolated endophytes (from lavender stem and leaves) cluster with already described* Pantoea *spp.; another group of isolates (all from the leaves) are diverging and thus may represent none yet described strains are found to be associated with plants.
- Figure 7 depicts the phylogenetic position of* Microbacterium* isolates (mainly from leaves and rhizosphere) in the context of the genus reference strains; noticeably many leaves isolates cluster with* M. hydrocarbonoxydans* and* M. oleivorans*, species whose members are able to perform crude oil degradation [49].
- Concerning* Bacillus* isolates (Suppl. Figure 1), a cluster of soil isolates including* Bacillus mojavensis *was disclosed. Another group, again comprising soil isolates, showed similarities with the stress resistant* B. safensis*.
- Lastly, more than the half of the bacterial isolates were affiliated to the genus* Pseudomonas;* the phylogenetic tree reported in Supplemental Figure 2 is quite complex. Regardless of the overall low support of the tree, (typical of* Pseudomonas* phylogenies), some observations may be drawn; the presence of large unresolved clusters shows clearly a low divergence of the isolated colonies, implying the existence of clonal populations;* Pseudomonas* spp. isolated from the different compartments are intermixed, suggesting the presence of a continuum rhizosphere-leaves (*Pseudomonas* is the only genus found in all the 4 sample analysed).

### 3.2. Inhibition of* Burkholderia Cepacia* Complex Strains Growth by* L. angustifolia* Endophytic Bacteria

In order to check the ability of* L. angustifolia* bacterial endophytes to antagonize the growth of (opportunistic) human pathogenic bacteria, cross-streak experiments were carried out as described in Section 2 using a panel of the 19 randomly chosen different endophytic strains isolated from soil, roots, or leafs and phylogenetically assigned to six different genera (*Bacillus*,* Microbacterium*,* Pantoea*,* Plantibacter*,* Pseudomonas*, and* Rhizobium*), as testers* versus* 40 Bcc strains representative of seventeen species (out of eighteen) and with either environmental or clinical origin, with some of which being multidrug-resistant. Data obtained are shown in Table 4. The analysis of these data revealed that all the tester strains were able to completely inhibit the growth of Bcc strains, including those with a clinical source and that exhibited multidrug resistance; this finding suggested that lavender endophytic bacteria are able to synthesize (strong) antimicrobial compounds. However, tester strains showed a different pattern of Bcc growth inhibition, even those affiliated to the same genus. In addition to this, there is no apparent difference in the antimicrobial potential between strains belonging to different plant compartments. Concerning the sensitivity of Bcc strains to the antimicrobial activity of lavender endophytes, strains belonging to different species exhibited a different sensitivity spectrum. However, it is quite interesting that strains with clinical origin appeared to be more sensitive to the antimicrobials synthesized by endophytic bacteria than their environmental counterparts (see for instance strains belonging to the species* Burkholderia cenocepacia* IIIB) (Supplementary Table 3).

## 4. Discussion

Medicinal plants are stirring the attention of many researchers, due to the presence of compounds that constitute a large fraction of the current pharmacopoeias. Natural products have been the source of most of the active ingredients of medicines and more than 80% of drug substances are natural products or inspired by a natural compound, including most of the of anticancer and anti-infective agents [50]. Lavender plants are grown mainly for the production of essential oil, which has antiseptic and anti-inflammatory properties. In spite of the relative harsh environment for bacterial colonization,* L. angustifolia* tissues were found to be rich in bacterial diversity. However, the endophytic community isolated from different plant compartments showed different levels of diversity, with the leaf showing the highest level and the stem the lowest (Table 1). It is noteworthy that the same level of diversity (regardless of community composition) showed by the leaf and roots community is already reported in other species [26] even if there are few direct comparisons of roots and phyllosphere bacterial communities, especially comparisons using material from the same plants. The microbial community residing in the phyllosphere is faced with a nutrient poor and variable environment that is characterized by fluctuating temperature, humidity, and UV radiation and so it is predicted to be more diverse than that within the rhizosphere, a more homeostatic environment. These findings, supported also by the similar bacterial titre, are in agreement with the idea that the source of inoculum for the leaf community (except for the vertically transferred via seeds) may be exclusive (e.g., aerosol, even if the process is not yet clarified, Bulgarelli et al. 2013) and not related to transmission from the roots. Moreover, the low diversity of the community isolated in the stem, dominated by clonally (Table 2) populations of* Pseudomonas*, could be related to the highly specific main tissue (phloem) present there, which could provide an homeostatic environment, rich in nutrient (sucrose contained in the phloem tissues) but poorer (if compared to leaf and roots) in secondary metabolites that may promote ecological niche differentiation.

Concerning the taxonomic community composition, different taxonomic profiles were found for endosphere and rhizosphere. More in detail the rhizosphere community showed quite similar diversity indexes if compared to the roots one, but with a different composition; as an example, members of the phylum Actinobacteria are completely absent from the root internal tissues that are largely dominated by Proteobacteria, while they are present in the soil. To explain this finding a two-step model has been proposed [16]: soil abiotic properties determine the structure of the initial bacterial communities that is then modified by rhizodeposits with plant roots cell wall features and released metabolites promoting the growth of organotrophic bacteria, thereby initiating a soil community shift. In the second step, convergent host genotype-dependent selection [51, 52] fine-tunes community profiles thriving on the rhizoplane and within plant roots.

Moreover, endosphere communities were different between plant organs (roots, stems, and leaves), even if the internal tissues are dominated, as already reported [25, 52–54] by members of the phylum Proteobacteria; an example, while rhizobia were isolated from root internal tissues, they are completely absent from stems and leaves (Figures 2 and 3); isolates belonging to the genus* Pantoea,* on the contrary, were isolated from aerial parts but not from root tissues. Hence, it is possible that plant might “select” specific taxa for entering inside its compartment. Such hypothesis is supported also by the pairwise differentiation values; the communities isolated from the different tissues, even those in physical continuity, are in fact strongly differentiated with the stem, representing a low diversity compartment separating the two high diversity organs roots and leaves. In this view it is noteworthy that in lavender only* Pseudomonas* represents a ubiquitous and abundant genus of both rhizosphere and endosphere (Figure 3). The analysis of intrageneric diversity pointed out also that* Pseudomonas *isolates belong to a low differentiated clonal population (Table 2 and Suppl. Figure 2), suggesting also that this component of the community either diffused inside the organs from a radical or foliar entry point or established during plants development, putatively from the seed inoculum. A similar consideration can be proposed for isolates of rhizobia but for the rhizosphere-root internal tissues continuum: a low taxonomically differentiated community from the soil entered (increasing its relative abundance) inside the plant organ. An entry point from the leaves is on the contrary consistent with the distribution of* Pantoea* isolates with their strong diversity (Table 2) suggesting a diversification in response to the high variable leaf environment. The isolates belonging to the genus* Stenotrophomonas *show an interesting distribution pattern (Figure 3); they are rare in the rhizosphere, abundant in the roots and in the leaves, and absent in the stem; this finding suggests a different entry point or a negative selection in the stem; the second explanation is supported by admixture of root and leaves isolates (Figure 4).

The detailed phylogenetic analyses of the isolates enabled us also to putatively ascribe some isolates to already described endophytic strains that can be targeted for functional characterisation; many* Microbacterium* leaves isolates cluster with* M. hydrocarbonoxydans* and* M. oleivorans*, two species with crude oil degrading activity [49] being this metabolic activity that is found frequently associated with a phyllosphere adapted lifestyle [55]. Several isolates cluster with bacteria with interesting biotechnological or ecological applications, such as* S. chelatiphaga*, a remarkable species with biotechnological potential in phytoremediation [56] and* S. rizophila *a plant associated bacterium with antifungal activity [57] or* Bacillus mojavensis* a bacteriumclosely related to* B. subtilis *and already reported having an endophytic lifestyle and showing an antagonistic role against plant pathogenic fungi [58]. On the contrary, many lavender* Pseudomonas* isolates cluster with known plant pathogen strains highlighting the sometimes subtle difference among pathogenic and endophytic lifestyle and with* Pantoea agglomerans *(and also with lavender isolates clustering withinthe* B. licheniformis* and* B. soronensis* groups), a known plant associated bacterium, and an opportunistic pathogen in immune-compromised human individuals drawing attention to the possible pathogenic action of endophytic bacteria in humans [59, 60].

The overall analysis of the dendrograms points out that the characterization of isolates from endophytic communities can lead to the identification (as an example for* Pantoea* and rhizobia) of not yet described strains that may represent untapped resources of bioactive compounds of agricultural or medical interest.

Even though it has not been still completely demonstrated, it cannot be excluded that the possibility that the qualiquantitative spectrum of bioactive metabolites in medical plants and their essential oils may be related also on the activity of bacterial endophytes as they may promote plant health and growth, elicit plant metabolism, or directly produce biotechnologically relevant compounds [36]. In this context, the characterization of cultivable bacterial communities is a fundamental step to this purpose since it paves the way to the possibility to build collections of isolates that may be phenotypically typed for important traits. In our opinion, data obtained in this work (Table 4 and Supplementary Tables 2 and 3) offers a preliminary but very promising example of the biotechnological potential of bacteria isolated from medicinal plants. In this pilot study in fact the majority of the tested endophytes showed to inhibit the growth of several (in some case all) human pathogenic strains belonging to the Bcc complexes that are known for their resistance to traditional antibiotics. These data are particularly interesting if correlated with the recent finding that essential oils from different medicinal plants are able to strongly (or completely) interfere with the growth of the same Bcc strains [61] and that bacterial endophytes isolated from plants of the same species exhibited the same bioactivity (Maida et al., in preparation). It is therefore possible that the therapeutic properties of the essential oil might reside also on bioactive compounds of microbial origin. We are completely aware that the determination of the correlation (eventually) existing between the composition of bacterial endophytic communities and the bioactivity of essential oils extracted from a medicinal plant would require the parallel analysis of the bacterial community and the essential oil activity extracted from the same plant. However, data obtained in this work and those from Maida et al. [62] support this idea.

It is also particularly intriguing, the idea that the antimicrobial activity of essential oils may reside on the action of multiple compounds, some of them produced or elicited by the microbiota, limiting the observed rapid evolution of human pathogenic bacteria resistant to single molecule antimicrobials.

The characterization of the heterotrophic aerobic endophytic bacterial community of* L. angustifolia *highlighted the existence of a diversified community between different organs/tissues that may be related to the coexistence of different sources of inoculum and/or a selection of the communities promoted by nutrient sand metabolites availability and antagonistic forces. Several not previously characterized strains have been isolated and molecularly typed, confirming that the analysis of the bacterial communities inhabiting extreme or unconventional environments may represent a proper strategy for the discovery of untapped sources of functional biodiversity and bioactive molecule production.

## Supplementary Material

Supplementary Figure 1: Bayesian dendrogram showing the relationships among the 16S rDNA sequences of 47 isolates belonging to the genus Bacillus and those of reference type strains. Posterior probability values are indicated at the node. Nodes are collapsed at 70% probability. LT = bacteria isolated from the rhizosphere, LR = bacteria isolated from the roots, LS = bacteria isolated from the stem.
Supplementary Figure 2: Bayesian dendrogram showing the relationships among the 16S rDNA sequences of isolates belonging to the genus *Pseudomonas* and those of reference type strains. Posterior probability values are indicated at the node. Nodes are collapsed at 70% probability. LT= bacteria isolated from the rhizosphere, LR = bacteria isolated from the roots, LS = bacteria isolated from the stem, LL = bacteria isolated from the leaves.
Supplementary Figure 3: Maximum parsimony dendrogram showing the relationships among the 16S rDNA sequences of 37 isolates belonging to the genus *Stenotrophomonas* and those of reference type strains. Bootstrap values are indicated at the node. LL= bacteria isolated from the leaves, LR = bacteria isolated from the roots (see Material and Methods and Suppl. Table 4 for details).
Supplementary Figure 4: Maximum parsimony dendrogram showing the relationships among the 16S rDNA sequences of 46 isolates belonging to the genus *Rhizobium* and those of reference type strains. Bootstrap values are indicated at each node. Nodes are collapsed at 70% probability. LT= bacteria isolated from the rhizosphere, LR = bacteria isolated from the roots (see Material and Methods and Suppl. Table 4 for details).
Supplementary Figure 5: Maximum parsimony dendrogram showing the relationships among the 16S rDNA sequences of 25 isolates belonging to the genus *Pantoea* and those of reference type strains. Bootstrap values are indicated at the node. Nodes are collapsed at 70% probability. LS = bacteria isolated from the stem, LL = bacteria isolated from the leaves (see Material and Methods and Suppl. Table 4 for details).
Supplementary Figure 6: Maximum parsimony dendrogram showing the relationships among the 16S rDNA sequences of 30 isolates belonging to the genus *Microbacterium* and those of reference type strains. Bootstrap values are indicated at the node. Nodes are collapsed at 70% probability. LT = bacteria isolated from the rhizosphere, LS = bacteria isolated from the stem, LL = bacteria isolated from the leaves (see Material and Methods and Suppl. Table 4 for details).
Supplementary Figure 7: Maximum parsimony dendrogram showing the relationships among the 16S rDNA sequences of 47 isolates belonging to the genus *Bacillus* and those of reference type strains. Bootstrap values are indicated at the node. Nodes are collapsed at 70% probability. LT = bacteria isolated from the rhizosphere, LR = bacteria isolated from the roots, LS = bacteria isolated from the stem (see Material and Methods and Suppl. Table 4 for details).
Supplementary Figure 8: Maximum parsimony dendrogram showing the relationships among the 16S rDNA sequences of isolates belonging to the genus *Pseudomonas* and those of reference type strains. Bootstrap values are indicated at the node. Nodes are collapsed at 70% probability. LT= bacteria isolated from the rhizosphere, LR = bacteria isolated from the roots, LS = bacteria isolated from the stem, LL = bacteria isolated from the leaves (see Material and Methods and Suppl. Table 4 for details).
Supplementary Figure 9: NJ dendrogram showing the relationships among the 16S rDNA sequences of 37 isolates belonging to the genus *Stenotrophomans* and those of reference type strains. Scale bars represent the Kimura-2 distance. Bootstrap values are indicated at the node. Nodes are collapsed at 70% probability. LL= bacteria isolated from the leaves, LR = bacteria isolated from the roots.
Supplementary Figure 10: NJ dendrogram showing the relationships among the 16S rDNA sequences of 46 isolates belonging to the genus *Rhizobium* and those of reference type strains. Scale bars represent the Kimura-2 distance. Bootstrap values are indicated at each node. Nodes are collapsed at 70% probability. LT= bacteria isolated from the rhizosphere, LR = bacteria isolated from the roots.
Supplementary Figure 11: NJ dendrogram showing the relationships among the 16S rDNA sequences of 25 isolates belonging to the genus *Pantoea* and those of reference type strains. Scale bars represent the Kimura-2 distance. Bootstrap values are indicated at the node. Nodes are collapsed at 70% probability. LS = bacteria isolated from the stem, LL = bacteria isolated from the leaves.
Supplementary Figure 12: NJ dendrogram showing the relationships among the 16S rDNA sequences of 30 isolates belonging to the genus *Microbacterium* and those of reference type strains. Scale bars represent the Kimura-2 distance. Bootstrap values are indicated at the node. Nodes are collapsed at 70% probability. LT = bacteria isolated from the rhizosphere, LS = bacteria isolated from the stem, LL = bacteria isolated from the leaves.
Supplementary Figure 13: NJ dendrogram showing the relationships among the 16S rDNA sequences of 47 isolates belonging to the genus *Bacillus* and those of reference type strains. Scale bars represent the Kimura-2 distance. Bootstrap values are indicated at the node. Nodes are collapsed at 70% probability. LT = bacteria isolated from the rhizosphere, LR = bacteria isolated from the roots, LS = bacteria isolated from the stem.
Supplementary Figure 14: NJ dendrogram showing the relationships among the 16S rDNA sequences of isolates belonging to the genus *Pseudomonas* and those of reference type strains. Scale bars represent the Kimura-2 distance. Bootstrap values are indicated at the node. Nodes are collapsed at 70% probability. LT= bacteria isolated from the rhizosphere, LR = bacteria isolated from the roots, LS = bacteria isolated from the stem, LL = bacteria isolated from the leaves.
Supplementary Table 1: List of bacterial endophytic strains used in this work as tester in the crossstreak experiments.
Supplementary Table 2: Number of Bcc target strains whose growth is inhibited by lavender bacterial endophytes.
Supplementary Table 3: Sensitivity spectrum exhibited by the 40 Bcc strains in the cross streak experiment vs a panel of lavender endophytic bacterial isolates.
Supplementary Table 4: Length of aligned matrices (bp), Variable and conserved characters number, Parsimony-informative characters, Consistency Index (CI), Retention Index (RI) and species used as outgroup of dendrograms obtained with Maximun Parsimony (see Materials and Methods for details).

## Figures and Tables

**Table 1:** Percentage distribution of bacterial phyla isolated from *L. angustifolia* roots, stem, leaves, and rhizospheric soil; standard diversity indexes built on genera distribution are also presented.

	Soil	Roots	Stem	Leaves
Proteobacteria	40	94	95	93
Firmicutes	44	6	3	—
Actinobacteria	16	—	2	7

**Richness**	7	6	5	8
**Evenness **	0.73	0.72	0.45	0.76
**Shannon index**	2.06	1.88	1.06	2.29

**Table 2:** Intragenus diversity analysis based on 16S rRNA gene sequence identity; mean identity of all versus all 16S rRNA gene sequences (for each genus); mean distance (Kimura 2 parameter); number of pairwise comparisons of 16S sequences with identity >97%, considered as threshold of species identity (in brackets the total number of comparisons and the percentage); number of OTU with at least 1 pairwise comparison with identity >97%.

Genus	Number of isolates	Mean identity among OTU (%)	Mean distance among OTU	Number of pairwise comparisons with identity >97%	Number of OTU with identity >97%
*Pseudomonas *	172	94.25	0.013	3840 (14878, 25.8%)	170
*Bacillus *	47	93.04	0.027	329 (1128, 29%)	46
Rhizobia	46	98.61	0.016	952 (1081, 88%)	46
*Stenotrophomonas *	37	95.34	0.023	415 (703, 59%)	37
*Microbacterium *	30	95.00	0.059	120 (465, 25%)	29
*Pantoea *	26	86.95	0.126	24 (351, 6.8%)	7

**Table 3:** The 40 *Burkholderia cepacia* complex strains used as targets in the cross streak experiments.

Target strain		
Strain	Species	Origin
FCF1	*B. cepacia *	CF
FCF3		

LMG17588	*B. multivorans *	ENV

FCF16	*B. cenocepacia* (IIIA)	CF
J2315		

FCF18	*B. cenocepacia* (IIIB)	CF
FCF20		
FCF23		
FCF24		
FCF27		
FCF29		
FCF30		
LMG16654		
C5424		
CEP511		
MVPC1/16		ENV
MVPC1/73		

LMG19230	*B. cenocepacia* (IIIC)	ENV
LMG19240		

FCF38	*B. cenocepacia* (IIID)	CF
LMG21462		

FCF41	*B. stabilis *	CF

FCF42	*B. vietnamiensis *	CF
TVV75		ENV

LMG18941	*B. dolosa *	CF
LMG18942		
LMG18943		

MCI7	*B. ambifaria *	ENV
LMG19467		CF
LMG19182		ENV

LMG16670	*B. anthina *	ENV

FCF43	*B. pyrrocina *	CF

LSED4	*B. lata *	CF

LMG24064	*B. latens *	CF

LMG24065	*B. diffusa *	CF

LMG23361	*B. contaminans *	AI

LMG24067	*B. seminals *	CF

LMG24068	*B. metallica *	CF

LMG24066	*B. arboris *	ENV

LMG24263	*B. ubonensis *	NI

Abbreviations: CF: strains isolated from cystic fibrosis patients; AI: strains isolated from animal infection; NI: strains isolated from nosocomial infection; ENV: environmental strain.

**Table 4:** Growth of 40 *Burkholderia cepacia* complex strains in the presence/absence of endophytic bacterial strains isolated from lavender.

Species	Strain	Origin	LL1	LL2	LL3	LL4	LL6	LL7	LL9	LL10	LS1	LS2	LS3	LS4	LS5	LS6	LR1	LR2	LR3	LR4	LR5	C-
*B. cepacia *	FCF 1	CF	−	−	−	−	−	−	−	−	−	−	−	−	−	−	−	−	−	−	−	+
*B. cepacia *	FCF 3	CF	+/−	−	−	−	−	−	−	−	−	−	−	−	−	−	−	−	−	−	−	+
*B. multivorans *	LMG 17588	Env	+	−	−	+	+/−	+/−	−	+/−	+	−	+	+/−	+/−	−	+	−	+	−	−	+
*B. cenocepacia *(IIIA)	FCF 16	CF	−	−	−	−	−	−	−	−	−	−	−	−	−	−	−	−	−	−	−	+
*B. cenocepacia *(IIIA)	J2315	CF	+	−	−	+/−	+/−	−	−	+/−	−	−	+	−	−	−	−	−	−	−	−	+
*B. cenocepacia *(IIIB)	FCF 18	CF	+	−	−	−	−	−	−	−	−	−	−	−	−	−	+/−	−	−	−	−	+
*B. cenocepacia *(IIIB)	FCF 20	CF	+	+	+	+	+	+/−	+	+/−	+/−	+/−	+/−	+	+/−	+	+/−	+	+	+	+	+
*B. cenocepacia *(IIIB)	FCF 23	CF	+	−	−	−	−	−	−	−	+/− −	−	+	+/−	−	−	+	−	−	−	−	+
*B. cenocepacia *(IIIB)	FCF 24	CF	+	−	−	−	−	−	−	−	+/−	−	+/−	−	−	−	+	−	−	−	−	+
*B. cenocepacia *(IIIB)	FCF 27	CF	+	−	−	−	−	−	−	−	+/−	−	−	−	−	−	−	−	−	−	−	+
*B. cenocepacia *(IIIB)	FCF 29	CF	+	−	−	+/−	+/−	−	−	+/−	−	−	−	−	−	−	+	−	+	−	−	+
*B. cenocepacia *(IIIB)	FCF 30	CF	+	−	−	+/−	+/−	−	−	+/−	+/−	−	−	−	−	−	+	−	+	−	−	+
*B. cenocepacia *(IIIB)	LMG 16654	CF	+	+/−	−	+	+/−	+	−	+	+	−	+	+	−	−	+	−	+	−	−	+
*B. cenocepacia *(IIIB)	C5424	CF	+	+/−	−	+/−	−	−	−	+/−	−	−	+/−	−	−	−	+/−	−	+	−	−	+
*B. cenocepacia *(IIIB)	CEP511	CF	+	−	−	+/−	−	−	−	+/−	+/−	−	+/−	+/−	−	−	+	−	+	−	−	+
*B. cenocepacia *(IIIB)	MVPC 1/16	Env	+	+/−	−	+/−	+	+/−	−	+/−	+/−	+/−	+/−	−	+/−	−	+/−	−	+	−	+/−	+
*B. cenocepacia *(IIIB)	MVPC 1/73	Env	+	+	+	+/−	+	+	+/−	+	+	+	+	+	+	+	+	+	+	+	+/−	+
*B. cenocepacia *(IIIIC)	LMG 19230	Env	+	+	+	+	+	+	+/−	+	+	+	+	+	+	+	+	+	+	+	+	+
*B. cenocepacia *(IIIC)	LMG 19240	Env	+	+	+	+	+/−	+	−	+	+	+/−	+	+	+	+/−	+	+	+	+	+/−	+
*B. cenocepacia *(IIID)	FCF 38	CF	+	−	−	−	−	−	−	−	−	−	−	−	−	−	−	−	−	−	−	+
*B. cenocepacia *(IIID)	LMG 21462	CF	−	−	−	−	−	−	−	−	−	−	−	−	−	−	−	−	−	−	−	+
*B. stabilis *	FCF 41	CF	+	+/−	+	+	+/−	+/−	−	+	+	+	−	+	+/−	−	+	+	+	+/−	−	+
*B. vietnamiensis *	FCF 42	CF	−	−	−	−	−	−	−	−	−	−	−	−	−	−	−	−	−	−	−	+
*B. vietnamiensis *	TVV 75	Env	+	+/−	−	+/−	−	−	−	+/−	−	−	+	+/−	−	−	+/−	−	+	−	−	+
*B. dolosa *	LMG 18941	CF	+	−	−	+/−	+/−	+/−	−	+/−	−	−	+	+/−	−	−	+/−	+/−	+	−	−	+
*B. dolosa *	LMG 18942	CF	+	*+ *	−	−	+/−	+/−	−	+	+	+/−	+	+/−	−	−	+	+/−	+	+/−	−	+
*B. dolosa *	LMG 18943	CF	+	+/−	−	+	+/−	+/−	−	+/−	+/−	−	+	+/−	−	−	+	+/−	+	−	−	+
*B. ambifaria *	MCI 7	Env	+	+	−	+/−	+/−	+/−	−	+	+/−	−	+	+/−	−	−	+	−	+	−	−	+
*B. ambifaria *	LMG 19467	CF	+	+	−	+/−	+/−	−	−	+	+/−	−	+	+/−	−	−	+	−	+/−	−	−	+
*B. ambifaria *	LMG 19182	Env	+	+	−	+/−	+/−	−	−	+	−	−	+	−	−	−	+	−	+/−	−	−	+
*B. anthina *	LMG 16670	Env	+	−	−	−	−	+	−	+	−	−	+	+/−	−	−	+	−	+/−	−	−	+
*B. pyrrocinia *	FCF 43	CF	+/−	−	−	−	−	−	−	−	−	−	+/−	−	−	−	−	−	+/−	−	−	+
*B. lata *	LSED 4	CF	+	−	*+ *	−	−	+/−	−	+	−	−	+	+/−	−	−	+/−	+/−	+/−	−	−	+
*B. latens *	LMG 24064	CF	+	−	*+ *	+/−	−	+/−	−	+	+	−	+	+	−	+	+	+	+	+	−	+
*B. diffusa *	LMG 24065	CF	+	+/−	*+ *	+/−	+/−	+	−	+	+	−	+	+	−	+/−	+	+	+	+	−	+
*B. contaminans *	LMG 23361	AI	+	+/−	+/−	+/−	+/−	+	−	+	+	+	+	+	+	+/−	+	+	+	+	−	+
*B. seminalis *	LMG 24067	CF	+	+/−	+	+/−	+	+	+	+	+	+	+	+	+	+	+	+	+	+	−	+
*B. metallica *	LMG 24068	CF	+	+	+	+/−	+	+	+	+	+	+	+	+	+	+	+	+	+	+	−	+
*B. arboris *	LMG 24066	Env	+	+/−	+	+/−	+	+	+/−	+	+	+	+	+	+/−	+/−	+	+	+	+/−	−	+
*B. ubonensis *	LMG 24263	NI	+	−	−	−	−	+/−	−	+/−	+	−	+	+	−	−	+/−	−	+/−	−	−	+

Abbreviations: CF: strains isolated from cystic fibrosis patients; AI: strains isolated from animal infection; NI: strains isolated from nosocomial infection; ENV: environmental strain; L: strains isolated from the leaf; S: strains isolated from the stem; R: strains isolated from the root.

Symbols: +: growth; +/−: reduced growth; +/− −: very reduced growth; −: no growth; C-: Petri dishes containing only the target strains.
