# Supplementary material for: Linking Bacterial Endophytic Communities to Essential Oils: Clues from Lavandula angustifolia Mill
Source: Evid Based Complement Alternat Med. 2014 May 26;2014:650905. doi: 10.1155/2014/650905 (PMC4058287; doi:10.1155/2014/650905)
Supplement: Supplementary file 1 — Supplementary Figure 1: Bayesian dendrogram showing the relationships among the 16S rDNA sequences of 47 isolates belonging to the genus Bacillus and those of reference type strains. Posterior probability values are indicated at the node. Nodes are collapsed at 70% probability. LT = bacteria isolated from the rhizosphere, LR = bacteria isolated from the roots, LS = bacteria isolated from the stem. Supplementary Figure 2: Bayesian dendrogram showing the relationships among the 16S rDNA sequences of isolates belonging to the genus Pseudomonas and those of reference type strains. Posterior probability values are indicated at the node. Nodes are collapsed at 70% probability. LT= bacteria isolated from the rhizosphere, LR = bacteria isolated from the roots, LS = bacteria isolated from the stem, LL = bacteria isolated from the leaves. Supplementary Figure 3: Maximum parsimony dendrogram showing the relationships among the 16S rDNA sequences of 37 isolates belonging to the genus Stenotrophomonas and those of reference type strains. Bootstrap values are indicated at the node. LL= bacteria isolated from the leaves, LR = bacteria isolated from the roots (see Material and Methods and Suppl. Table 4 for details). Supplementary Figure 4: Maximum parsimony dendrogram showing the relationships among the 16S rDNA sequences of 46 isolates belonging to the genus Rhizobium and those of reference type strains. Bootstrap values are indicated at each node. Nodes are collapsed at 70% probability. LT= bacteria isolated from the rhizosphere, LR = bacteria isolated from the roots (see Material and Methods and Suppl. Table 4 for details). Supplementary Figure 5: Maximum parsimony dendrogram showing the relationships among the 16S rDNA sequences of 25 isolates belonging to the genus Pantoea and those of reference type strains. Bootstrap values are indicated at the node. Nodes are collapsed at 70% probability. LS = bacteria isolated from the stem, LL = bacteria isolated from the leaves (see Mat [file 650905.f1.pdf]

Supplementary Figure 1. Bayesian dendrogram showing the relationships among the 16S rDNA sequences of 47 isolates belonging to the genus *Bacillus* and those of reference type strains. Posterior probability values are indicated at the node. Nodes are collapsed at 70% probability. LT = bacteria isolated from the rhizosphere, LR = bacteria isolated from the roots, LS = bacteria isolated from the stem.

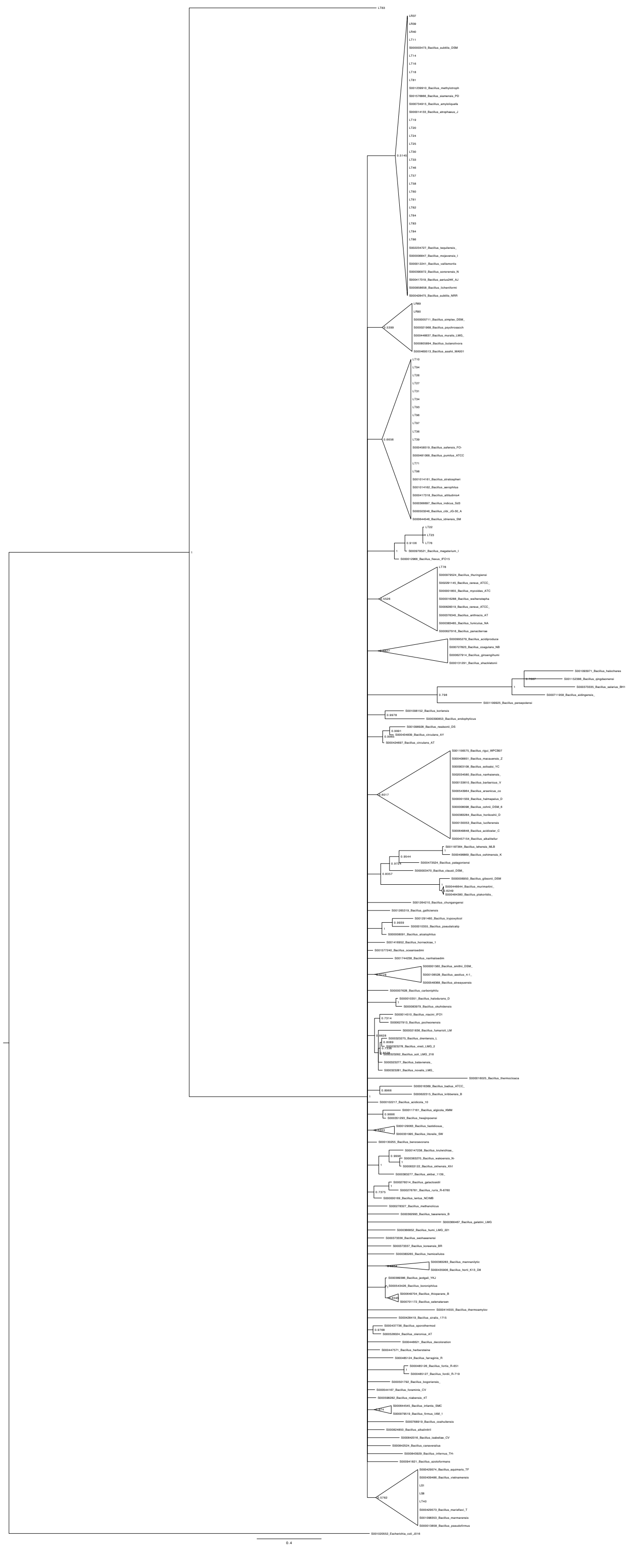

Supplementary Figure 2. Bayesian dendrogram showing the relationships among the 16S rDNA sequences of isolates belonging to the genus *Pseudomonas* and those of reference type strains. Posterior probability values are indicated at the node. Nodes are collapsed at 70% probability. LT= bacteria isolated from the rhizosphere, LR = bacteria isolated from the roots, LS = bacteria isolated from the stem, LL = bacteria isolated from the leaves.

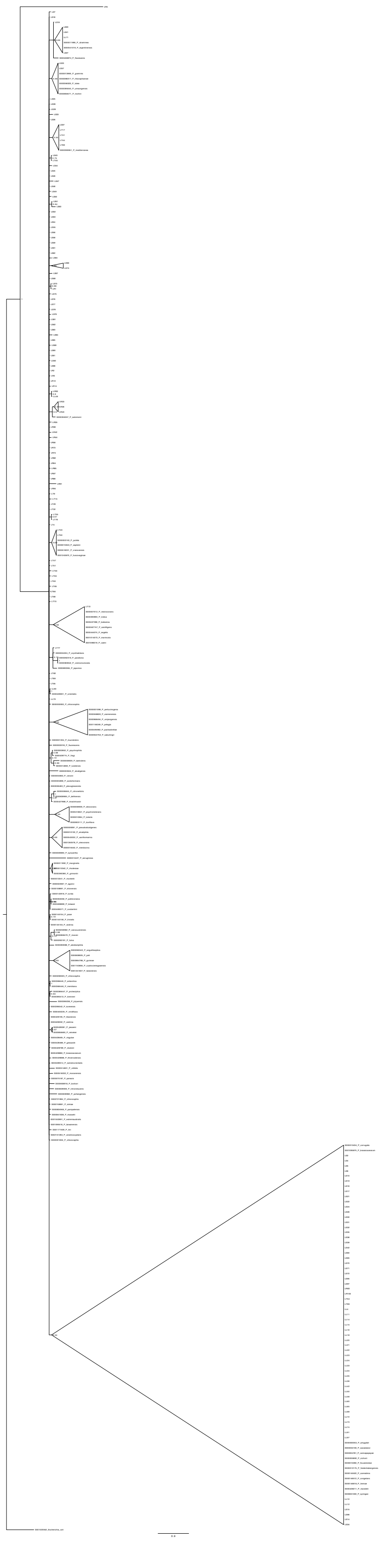

Supplementary Figure 3. Maximum parsimony dendrogram showing the relationships among the 16S rDNA sequences of 37 isolates belonging to the genus *Stenotrophomonas* and those of reference type strains. Bootstrap values are indicated at the node. LL= bacteria isolated from the leaves, LR = bacteria isolated from the roots (see Material and Methods and Suppl. Table 4 for details).

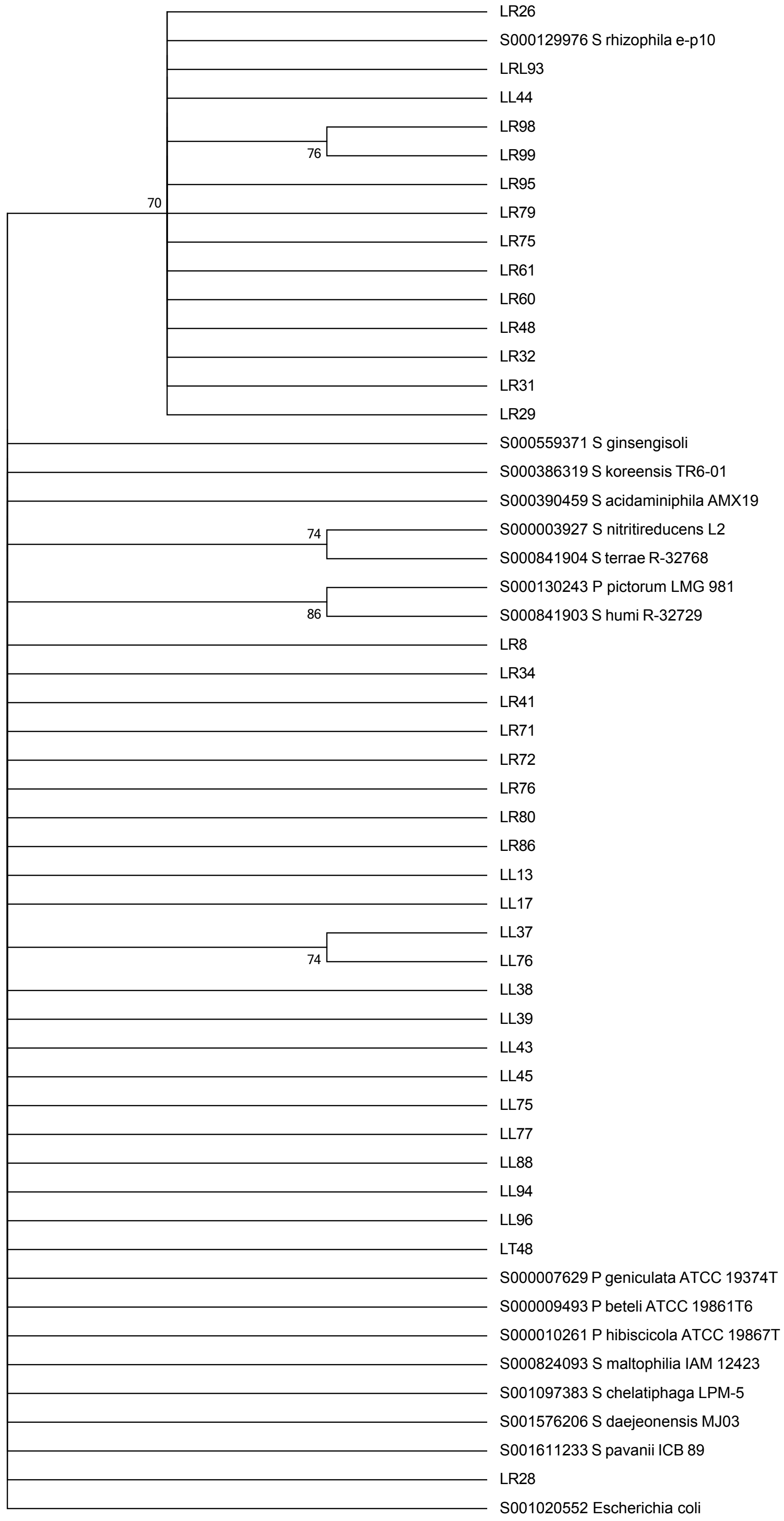

Supplementary Figure 4. Maximum parsimony dendrogram showing the relationships among the 16S rDNA sequences of 46 isolates belonging to the genus *Rhizobium* and those of reference type strains. Bootstrap values are indicated at each node. Nodes are collapsed at 70% probability. LT= bacteria isolated from the rhizosphere, LR = bacteria isolated from the roots (see Material and Methods and Suppl. Table 4 for details).

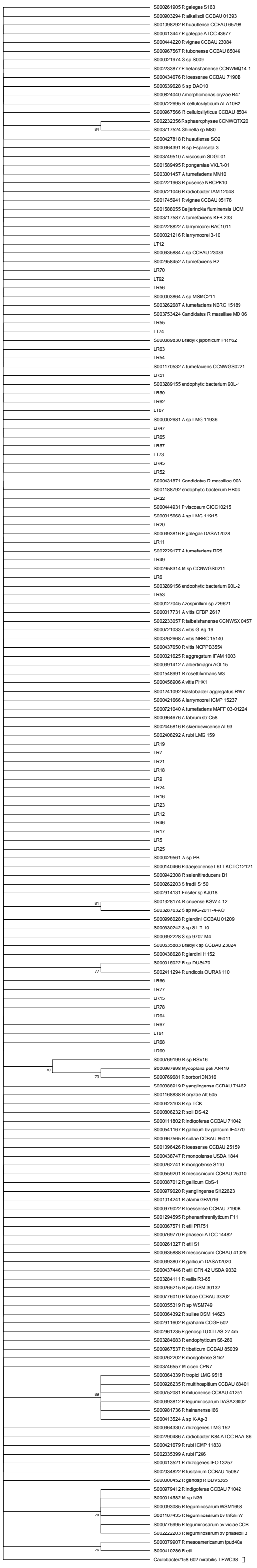

Supplementary Figure 5. Maximum parsimony dendrogram showing the relationships among the 16S rDNA sequences of 25 isolates belonging to the genus *Pantoea* and those of reference type strains. Bootstrap values are indicated at the node. Nodes are collapsed at 70% probability. LS = bacteria isolated from the stem, LL = bacteria isolated from the leaves (see Material and Methods and Suppl. Table 4 for details).

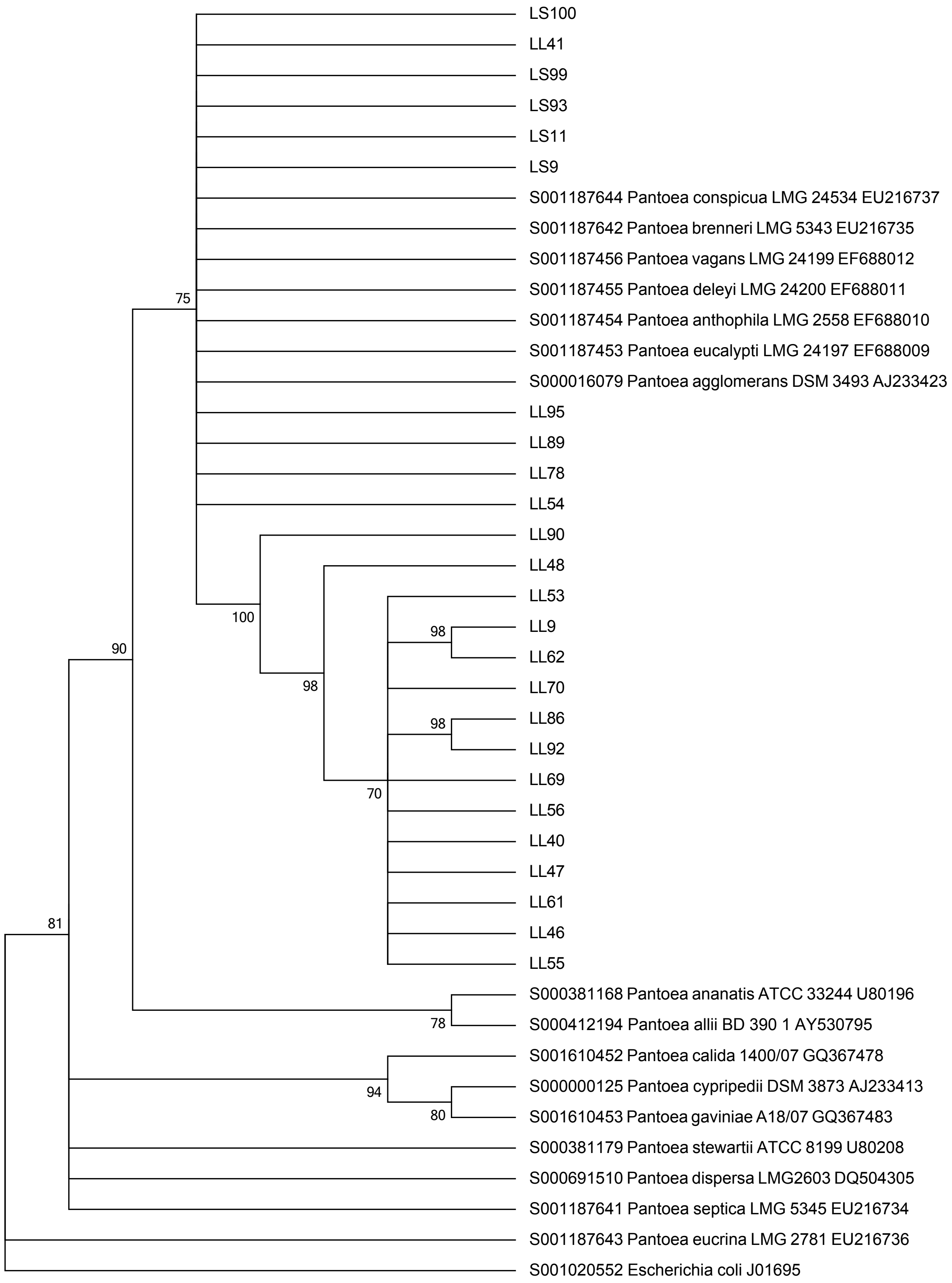

Supplementary Figure 6. Maximum parsimony dendrogram showing the relationships among the 16S rDNA sequences of 30 isolates belonging to the genus *Microbacterium* and those of reference type strains. Bootstrap values are indicated at the node. Nodes are collapsed at 70% probability. LT = bacteria isolated from the rhizosphere, LS = bacteria isolated from the stem, LL = bacteria isolated from the leaves (see Material and Methods and Suppl. Table 4 for details).

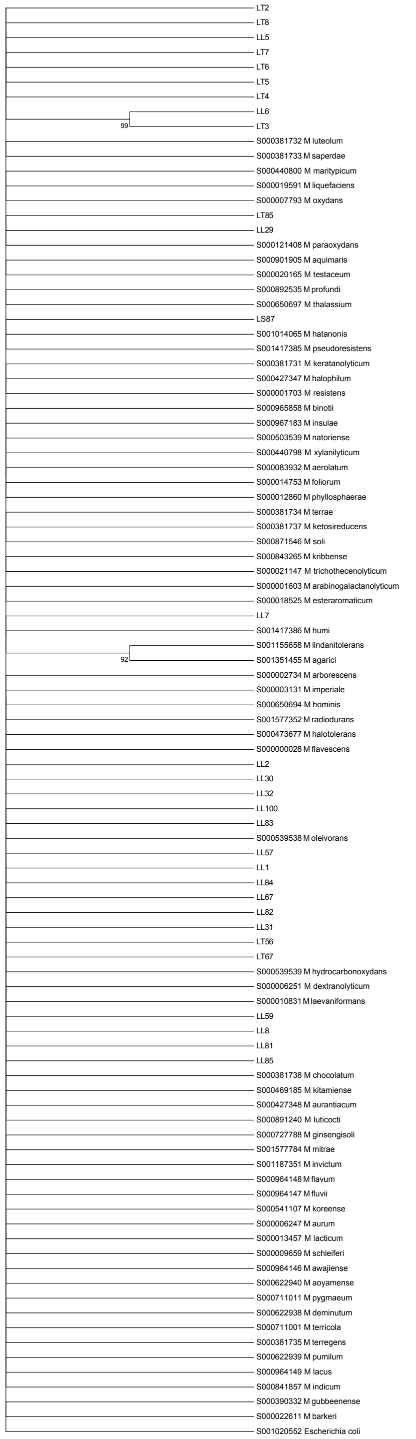

Supplementary Figure 7. Maximum parsimony dendrogram showing the relationships among the 16S rDNA sequences of 47 isolates belonging to the genus *Bacillus* and those of reference type strains. Bootstrap values are indicated at the node. Nodes are collapsed at 70% probability. LT = bacteria isolated from the rhizosphere, LR = bacteria isolated from the roots, LS = bacteria isolated from the stem (see Material and Methods and Suppl. Table 4 for details).

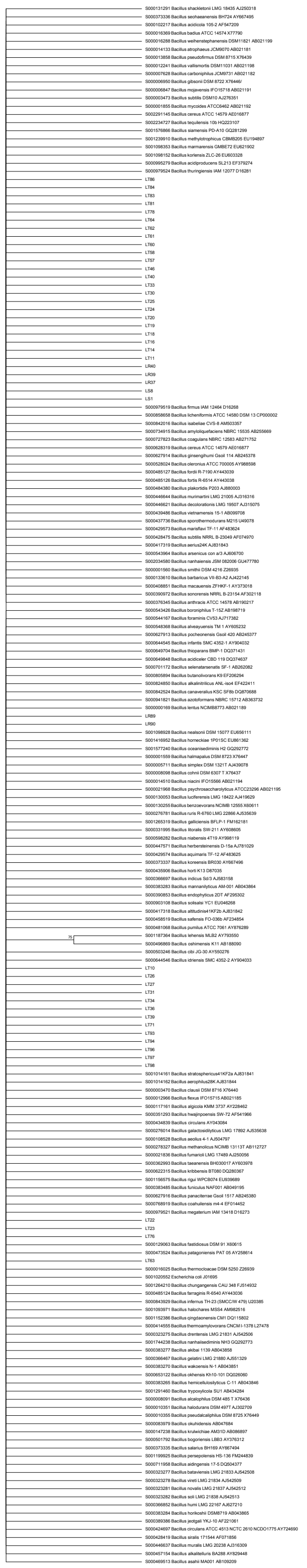

Supplementary Figure 8. Maximum parsimony dendrogram showing the relationships among the 16S rDNA sequences of isolates belonging to the genus *Pseudomonas* and those of reference type strains. Bootstrap values are indicated at the node. Nodes are collapsed at 70% probability. LT= bacteria isolated from the rhizosphere, LR = bacteria isolated from the roots, LS = bacteria isolated from the stem, LL = bacteria isolated from the leaves (see Material and Methods and Suppl. Table 4 for details).

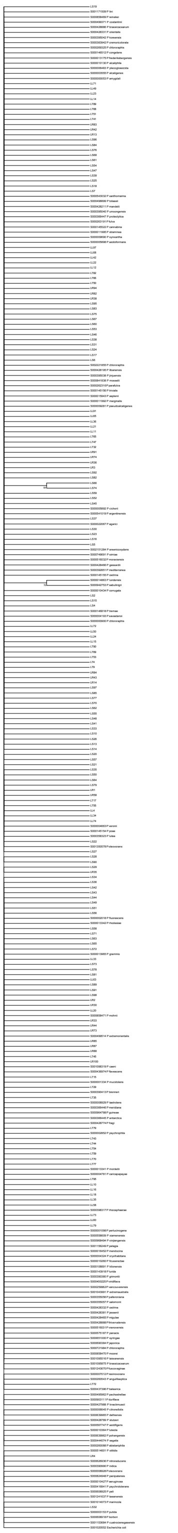

Supplementary Figure 9. NJ dendrogram showing the relationships among the 16S rDNA sequences of 37 isolates belonging to the genus *Stenotrophomans* and those of reference type strains. Scale bars represent the Kimura-2 distance. Bootstrap values are indicated at the node. Nodes are collapsed at 70% probability. LL= bacteria isolated from the leaves, LR = bacteria isolated from the roots.

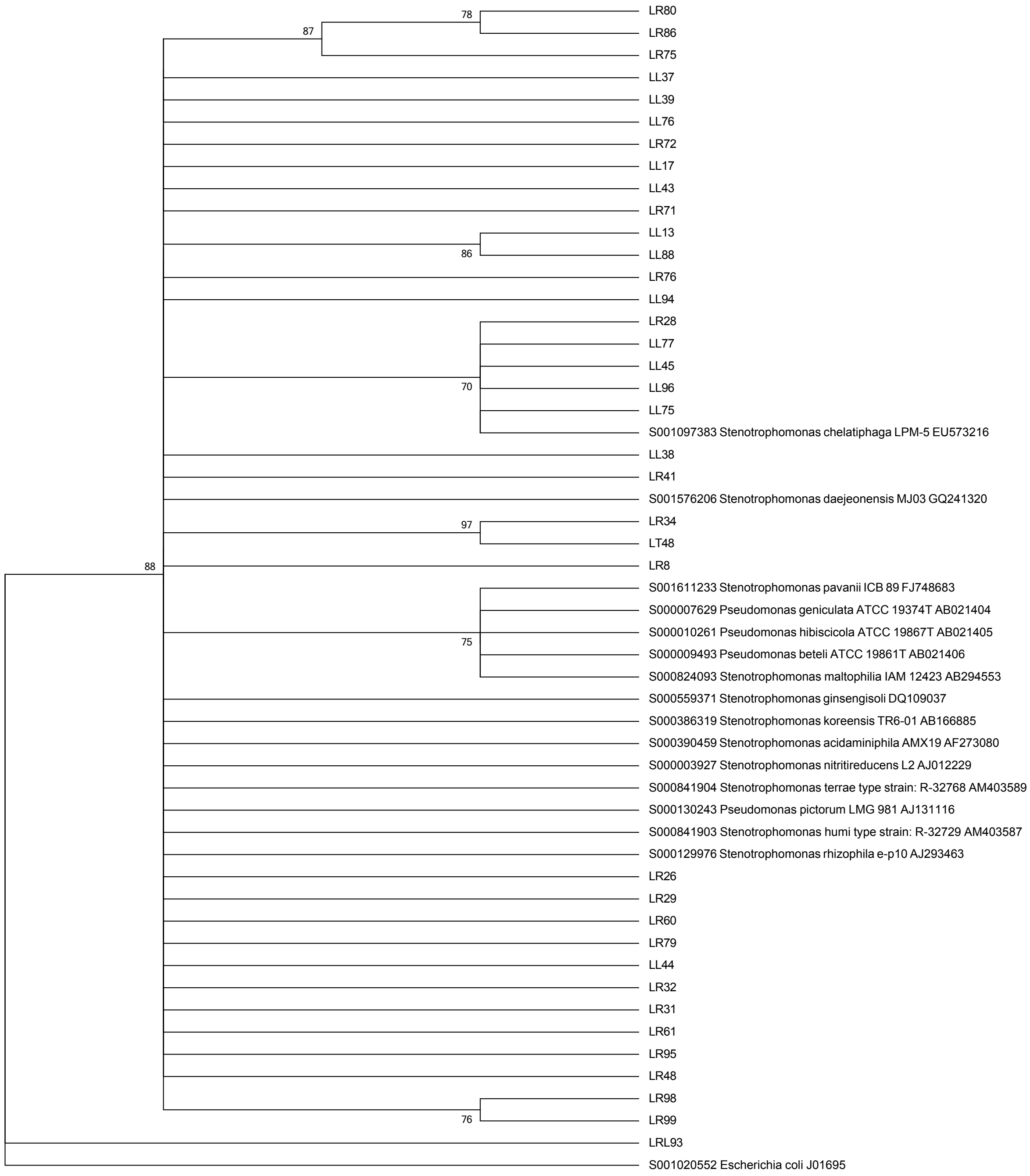

Supplementary Figure 10. NJ dendrogram showing the relationships among the 16S rDNA sequences of 46 isolates belonging to the genus *Rhizobium* and those of reference type strains. Scale bars represent the Kimura-2 distance. Bootstrap values are indicated at each node. Nodes are collapsed at 70% probability. LT= bacteria isolated from the rhizosphere, LR = bacteria isolated from the roots.

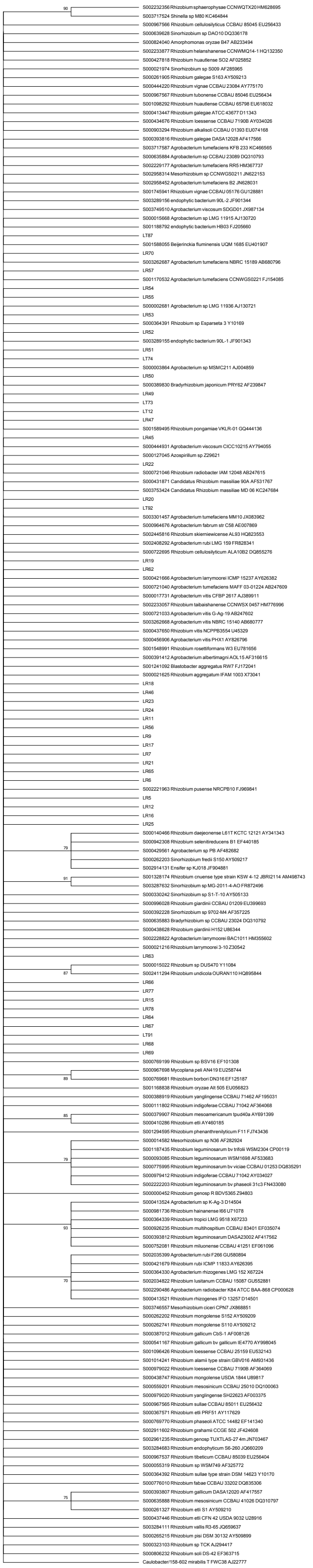

Supplementary Figure 11. NJ dendrogram showing the relationships among the 16S rDNA sequences of 25 isolates belonging to the genus *Pantoea* and those of reference type strains. Scale bars represent the Kimura-2 distance. Bootstrap values are indicated at the node. Nodes are collapsed at 70% probability. LS = bacteria isolated from the stem, LL = bacteria isolated from the leaves.

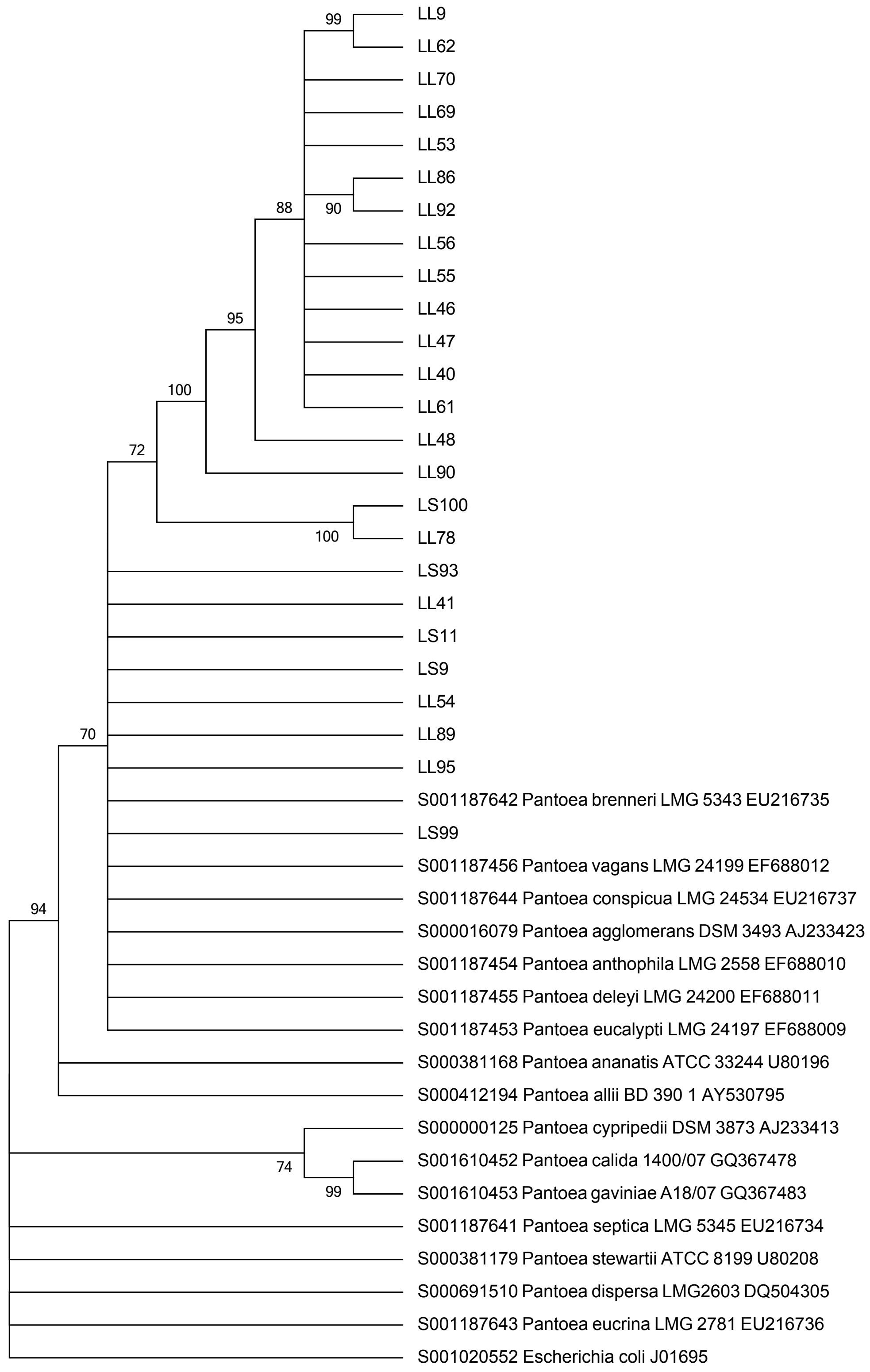

Supplementary Figure 12. NJ dendrogram showing the relationships among the 16S rDNA sequences of 30 isolates belonging to the genus *Microbacterium* and those of reference type strains. Scale bars represent the Kimura-2 distance. Bootstrap values are indicated at the node. Nodes are collapsed at 70% probability. LT = bacteria isolated from the rhizosphere, LS = bacteria isolated from the stem, LL = bacteria isolated from the leaves.

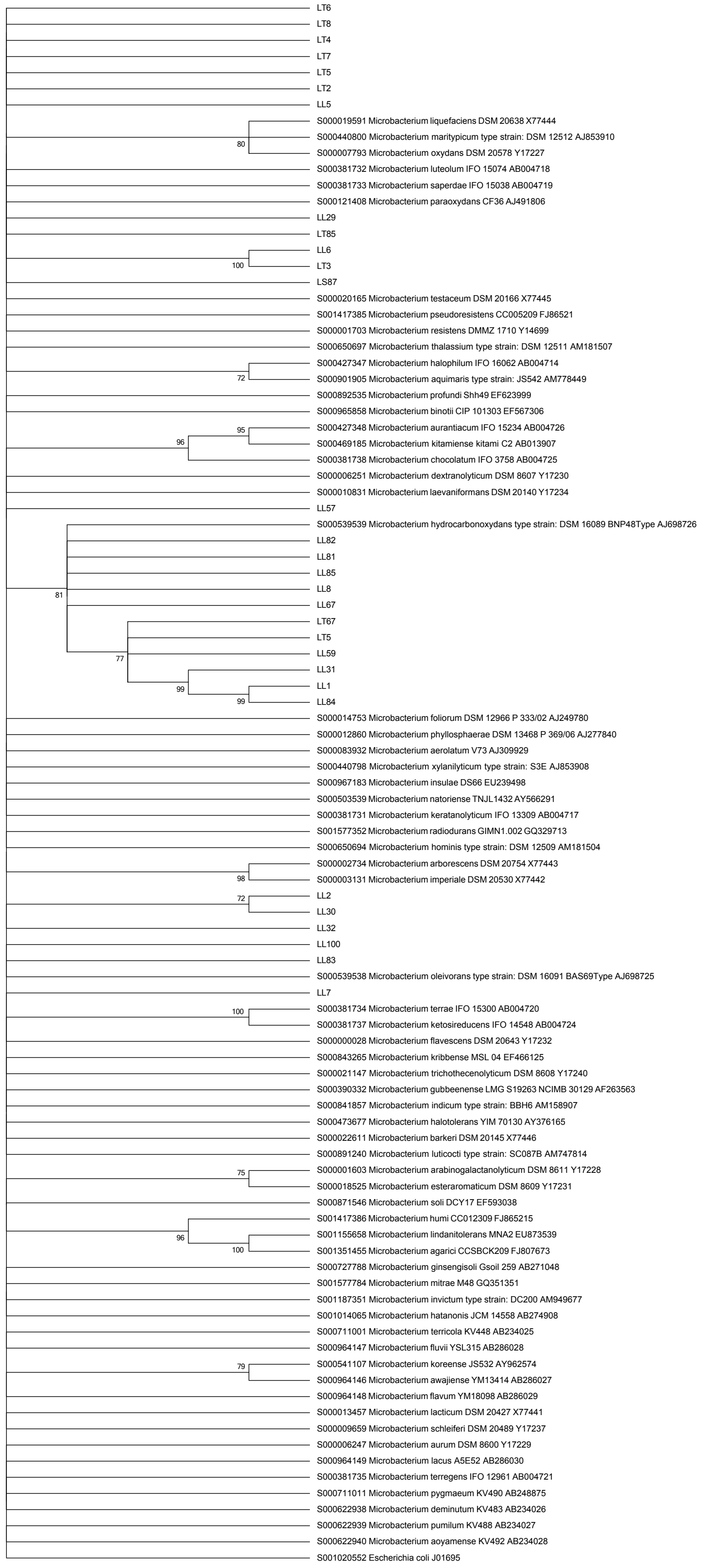

Supplementary Figure 13. NJ dendrogram showing the relationships among the 16S rDNA sequences of 47 isolates belonging to the genus *Bacillus* and those of reference type strains. Scale bars represent the Kimura-2 distance. Bootstrap values are indicated at the node. Nodes are collapsed at 70% probability. LT = bacteria isolated from the rhizosphere, LR = bacteria isolated from the roots, LS = bacteria isolated from the stem.

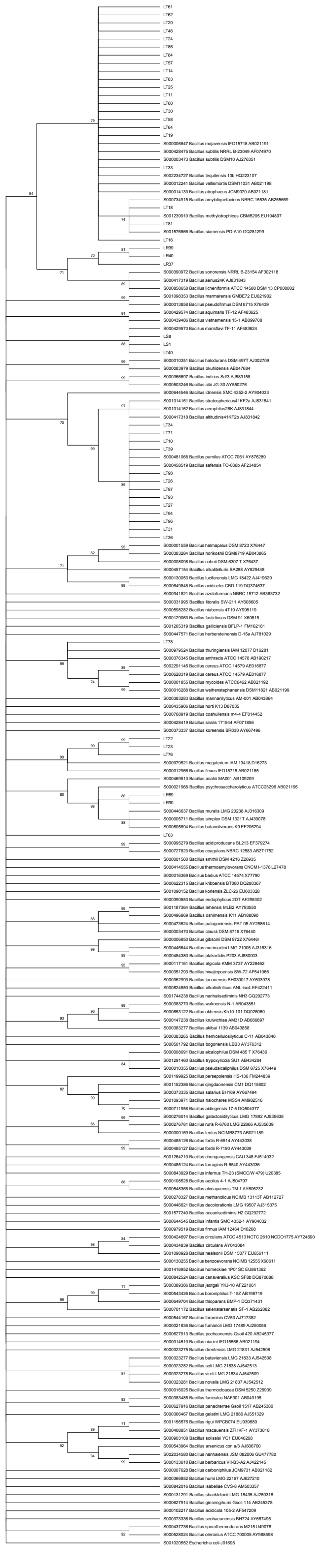

Supplementary Figure 14. NJ dendrogram showing the relationships among the 16S rDNA sequences of isolates belonging to the genus *Pseudomonas* and those of reference type strains. Scale bars represent the Kimura-2 distance. Bootstrap values are indicated at the node. Nodes are collapsed at 70% probability. LT= bacteria isolated from the rhizosphere, LR = bacteria isolated from the roots, LS = bacteria isolated from the stem, LL = bacteria isolated from the leaves.

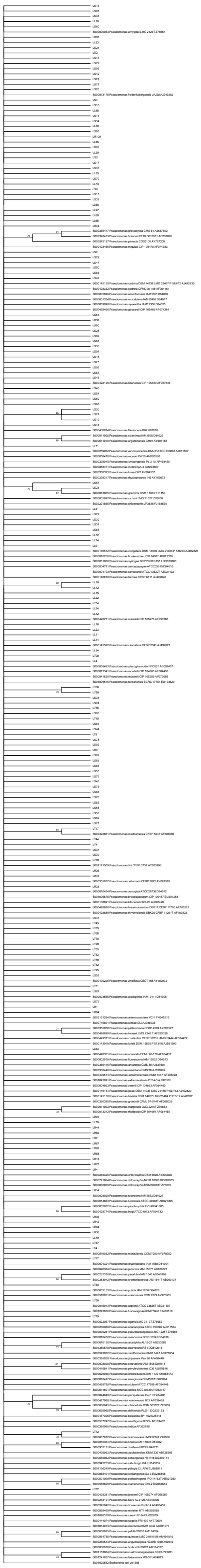

Supplementary Table 1. List of bacterial endophytic strains used in this work as tester in the cross-streak experiments.

**Isolate**	**Genus**	**Origin**
LL1	*Microbacterium*	Leaf
LL2	*Microbacterium*	Leaf
LL3	*Plantibacter*	Leaf
LL4	*Pseudomonas*	Leaf
LL6	*Microbacterium*	Leaf
LL7	*Microbacterium*	Leaf
LL9	*Pantoea*	Leaf
LL10	*Pseudomonas*	Leaf
LS1	*Bacillus*	Stem
LS2	*Pseudomonas*	Stem
LS3	*Bacillus*	Stem
LS4	*Pseudomonas*	Stem
LS5	*Pseudomonas*	Stem
LS6	*Pseudomonas*	Stem
LR1	*Pseudomonas*	Root
LR2	*Pseudomonas*	Root
LR3	*Pseudomonas*	Root
LR4	*Pseudomonas*	Root
LR5	*Rizhobium*	Root

Abbreviations: LL, LS, LR, strains isolated from leaf; stem, and root, respectively.

Supplementary Table 2. Number of Bcc target strains whose growth is inhibited by lavender bacterial endophytes.

**Endophytic isolate**	**Genus**	**Number of Bcc strains inhibited**
LL1	*Microbacterium*	5
LL2	*Microbacterium*	21
LL3	*Plantibacter*	28
LL4	*Pseudomonas*	16
LL6	*Microbacterium*	23
LL7	*Microbacterium*	20
LL9	*Pantoea*	35
LL10	*Pseudomonas*	15
LS1	*Bacillus*	21
LS2	*Pseudomonas*	30
LS3	*Bacillus*	12
LS4	*Pseudomonas*	16
LS5	*Pseudomonas*	29
LS6	*Pseudomonas*	30
LR1	*Pseudomonas*	10
LR2	*Pseudomonas*	21
LR3	*Pseudomonas*	11
LR4	*Pseudomonas*	25
LR5	*Rhizobium*	36

Abbreviations: LL, LS, LR: strains isolated from leaf, stem and root, respectively.

Supplementary Table 3. Sensitivity spectrum exhibited by the 40 Bcc strains in the cross streak experiment vs a panel of lavender endophytic bacterial isolates.

N°	Species	Strain	Origin	Inhibited by
1	*B. cepacia (I)*	FCF 1	CF	All tested endophytes
2	*B. cepacia (I)*	FCF 3	CF	All tested endophytes
3	*B. multivorans (II)*	LMG 17588	ENV	All tested endophytes except for LL1, LL4, LS1, LS3, LR1, LR3
4	*B. cenocepacia (IIIA)*	FCF 16	CF	All tested endophytes
5	*B. cenocepacia (IIIA)*	J2315	CF	All tested endophytes except for LL1, LS3,
6	*B. cenocepacia (IIB)*	FCF 18	CF	All tested endophytes except for LL1
7	*B. cenocepacia (IIB)*	FCF 20	CF	Partially by LS1, LS2, LS3, LS5, LR1
8	*B. cenocepacia (IIB)*	FCF 23	CF	All tested endophytes except for LL1, LS3, LR1
9	*B. cenocepacia (IIB)*	FCF 24	CF	All tested endophytes except for LL1, LR1
10	*B. cenocepacia (IIB)*	FCF 27	CF	All tested endophytes except for LL1
11	*B. cenocepacia (IIB)*	FCF 29	CF	All tested endophytes except for LL1, LR1, LR3
12	*B. cenocepacia (IIB)*	FCF 30	CF	All tested endophytes except for LL1, LR1, LR3
13	*B. cenocepacia (IIB)*	LMG 16654	CF	LL2, LL3, LL6, LL9, LS2, LS5, LS6, LR2, LR4, LR5
14	*B. cenocepacia (IIB)*	C5424	CF	LL3, LL6, LL7, LL9, LS1, LS2, LS4, LS5, LS6
15	*B. cenocepacia (IIB)*	CEP511	CF	LL2, LL3, LL6, LL7, LL9, LS2, LS5, LS6
16	*B. cenocepacia (IIB)*	MVPC 1/16	ENV	LL3, LL9, LS4, LS6, LR2, LR4
17	*B. cenocepacia (IIB)*	MVPC 1/73	ENV	LL3, LL9, LR5
18	*B. cenocepacia (IIIC)*	LMG 19230	ENV	LL9, LR5
19	*B. cenocepacia (IIIC)*	LMG 19240	ENV	LL6, LL9, LS2, LS6, LR5
20	*B. cenocepacia (IIID)*	FCF 38	CF	All tested endophytes except for LL1
21	*B. cenocepacia (IIID)*	LMG 21462	CF	All tested endophytes
22	*B. stabilis (IV)*	FCF 41	CF	LL9, LS3, LS6, LR5
23	*B. vietnamiensis (V)*	FCF 42	CF	All tested endophytes
24	*B. vietnamiensis (V)*	TVV 75	ENV	LL3, LL6, LL7, LL9, LS1, LS2, LS5, LS6, LR2, LR4, LR5
25	*B. dolosa (VI)*	LMG 18941	CF	LL2, LL3, LL9, LS1, LS2, LS5, LS6, LR4, LR5
26	*B. dolosa (VI)*	LMG 18942	CF	LL3, LL9, LS5, LS6, LR5
27	*B. dolosa (VI)*	LMG 18943	CF	LL3, LL9, LS2, LS5, LS6, LR4, LR5
28	*B. ambifaria (VII)*	MCI 7	ENV	LL3, LL9, LS2, LS5, LS6, LR2, LR4, LR5
29	*B. ambifaria (VII)*	LMG 19467	CF	LL3, LL7, LL9, LS2, LS5, LS6, LR2, LR4, LR5
30	*B. ambifaria (VII)*	LMG 19182	ENV	LL3, LL7, LL9, LS1, LS2, LS4, LS5, LS6, LR2, LR4, LR5
31	*B. anthina (VIII)*	LMG 16670	ENV	LL2, LL3, LL4, LL6, LL9, LS1, LS2, LS5, LS6, LR2, LR4, LR5
32	*B. pyrrocinia (IX)*	FCF 43	CF	LL2, LL3, LL4, LL6, LL7, LL9, LL10, LS1, LS2, LS5, LS6, LR4, LR5
33	*B. lata*	LSED 4	CF	LL2, LL4, LL6, LL9, LS1, LS2, LS5, LS6, LRA, LR5
34	*B. latens*	LMG 24064	CF	LL2, LL6, LL9, LS2, LS5, LR5
35	*B. diffusa*	LMG 24065	CF	LL9, LS2, LS5, LR5
36	*B. contaminans*	LMG 23361	AI	LL9, LR5
37	*B. seminalis*	LMG 24067	CF	LR5
38	*B. metallica*	LMG 24068	CF	LR5
39	*B. arboris*	LMG 24066	ENV	LR5
40	*B. ubonensis*	LMG 24263	NI	LR5

Abbreviations: CF, strains isolated from Cystic Fibrosis patients; AI, strains isolated from Animal Infection; NI, strains isolated from Nosocomial Infection; ENV, environmental strain; LL, LS, LR: strains isolated from leaf, stem, and root, respectively.

Supplementary Table 4. Length of aligned matrices (bp), Variable and conserved characters number, Parsimony-informative characters, Consistency Index (CI), Retention Index (RI) and species used as outgroup of dendrograms obtained with Maximun Parsimony (see Materials and Methods for details).

**Dendrogram**	**Aligned matrix length (bp)**	**Conserved characters**	**Variable characters**	**Parsimony informative characters**	**Retention Index**	**Consistency Index**	**Species used as outgroup**
Pantoea	545	344	201	161	0.9487	0.6383	*E. coli*
Pseudomonas	610	175	423	210	0.9717	0.4434	*E. coli*
Bacillus	619	281	327	227	0.9241	0.1904	*E. coli*
Rhizobium	458	296	152	75	0.9593	0.4379	*C. mirabilis*
Stenotrophomonas	524	314	192	89	0.9707	0.6874	*E. coli*
Microbacterium	566	337	199	111	0.9522	0.3379	*E. coli*
